# Food Insecurity and Academic Performance among Postsecondary Students: A Global Scoping Review

**DOI:** 10.1016/j.cdnut.2026.109551

**Published:** 2026-10-06

**Authors:** Edris Afsharkohan, Alireza Majidi, Mahdi Hejazi Laein, Ginny Lane, Mojtaba Shafiee

**Affiliations:** 1 College of Education, University of Findlay, Findlay, OH, United States; 2 Margaret Ritchie School of Family and Consumer Sciences, College of Agricultural and Life Sciences, University of Idaho, Moscow, ID, United States; 3 Department of Health Sciences, Faculty of Applied Health Sciences, Brock University, St. Catharines, ON, Canada; 4 School of Public Health, University of Saskatchewan, Saskatoon, SK, Canada

**Keywords:** food security, higher education, university students, college students, academic achievement, grade point average, scoping review, student success

## Abstract

Food insecurity is increasingly recognized as a significant public health concern among postsecondary students worldwide. Emerging evidence suggests that inadequate access to food may negatively affect academic success; however, the scope and consistency of this relationship have not been comprehensively synthesized. This scoping review aimed to systematically identify, map, and summarize the existing literature on the relationship between food insecurity and academic performance among postsecondary students worldwide. The review was conducted in accordance with the Preferred Reporting Items for Systematic Reviews and Meta-Analyses extension for Scoping Reviews guidelines. Six electronic databases (PubMed, Web of Science, Embase, CINAHL, PsycINFO, and ERIC) were searched from inception to November 2025. Eligibility criteria were defined using the Population, Concept, Context framework. Data were extracted and synthesized descriptively. Of 3078 records identified, 86 studies met the inclusion criteria, including 67 quantitative studies, 11 qualitative studies, and 8 mixed-methods studies. Most studies were conducted in the United States (*n* = 69). Across study designs, food insecurity was consistently associated with poorer academic outcomes, including lower grade point average, reduced academic achievement, impaired concentration, lower class attendance and engagement, increased course withdrawal and failure, delayed graduation, and lower educational attainment. Several studies identified anxiety, depression, psychological undefined distress, sleep disturbances, and financial strain as potential mechanisms linking food insecurity to academic performance. Qualitative evidence highlighted students’ experiences of hunger, fatigue, stress, competing financial demands, and work–study disagreement as barriers to academic success. The available evidence consistently indicates that food insecurity is associated with poorer academic performance among postsecondary students. Addressing food insecurity is a strategic opportunity to support student success, retention, and educational attainment. Additional longitudinal and intervention studies are needed to strengthen causal inference and identify effective approaches to improving both food security and academic outcomes in higher education settings.

## Introduction

Food insecurity, defined as inadequate or uncertain access to sufficient, safe, and nutritious food due to financial or other resource constraints [1], has emerged as a significant public health concern among postsecondary students [2], [3]. For nearly 2 decades, studies have documented food insecurity among college and university students [4], [5], [6], [7]. Early evidence from a survey conducted in 2006 at the University of Hawai‘i at Mānoa found that 21% of students experienced food insecurity [7]. More recent studies have continued to document high rates of food insecurity among postsecondary students that often exceed those observed in the general population [4], [5], [6], [7]. For example, a systematic review of food insecurity at higher education institutions in the United States reported an average prevalence of 43.5% among students compared with 13% among United States households in 2015 [5]. Other comprehensive syntheses have found that prevalence of food insecurity among college students in the United States varies widely, ranging from 10% to 75%, which reflects differences in study populations, measurement tools, and survey methodologies [6]. A systematic review and meta-analysis of postsecondary students in high-income countries found that food insecurity affected an estimated 42.2% of students [95% confidence interval (CI): 38.8%, 45.8%], with prevalence ranging from 11.8% to 98% across studies [8]. Similar patterns have been observed in other parts of the world. In Australia, food insecurity prevalence ranged from 38% to 48% [9], [10], and most recently ≤53% in 2024 [11]. Around the world, various countries have reported high prevalences of food insecurity among postsecondary students, including Canada at 42.3% [12], Germany at 27.5% [13], Turkey at 49.7% [14], and Lebanon at 39% [15]. These findings demonstrate that food insecurity among postsecondary students is a widespread global issue. Rising tuition costs, increasing living costs, limited financial resources, inadequate social support, lack of food and nutrition skills, and insufficient awareness of assistance programs have contributed to growing concerns regarding students’ ability to consistently access adequate food while pursuing higher education [2], [3], [16].

Food insecurity during postsecondary education is particularly concerning because it occurs during a critical developmental period characterized by substantial academic, financial, and social demands [17]. In addition to food security’s well-established associations with poorer dietary intake, physical health [18], and mental health outcomes [19], it may also compromise students’ ability to engage fully in their educational experiences [2], [20] and achieve academic success as indicated by grade point average (GPA), academic progression, course completion, retention, and graduation [21], [22]. Factors that undermine academic performance can have lasting implications for students’ educational trajectories, career opportunities, economic stability, and long-term well-being [23].

A growing body of research suggests that food insecurity may be an important determinant of academic success among postsecondary students [24], [25], [26], [27], [28], [29], [30]. Food insecurity has been linked to lower GPA [24], [25], reduced concentration [26], increased absenteeism [27], poor classroom engagement [28], delayed graduation [29], and lower educational attainment [30]. Qualitative research further suggests that students experiencing food insecurity often struggle to balance basic needs with academic responsibilities, leading to difficult trade-offs between purchasing food, paying educational expenses, working additional hours, and engaging in academic activities [31], [32], [33].

Despite the increasing attention devoted to college food insecurity, the evidence examining its relationship with academic performance remains fragmented. Studies have been conducted across a wide range of countries (e.g., United States [24], Canada [34], Australia [35], Jordan [36], Saudi Arabia [37], and Mexico [38]), institutional settings (e.g., public [28], private [39], and community college [40]), and student populations (e.g., undergraduate [41], postgraduate [36], international [42], and student veterans [40]) and have employed diverse methodological approaches (e.g., cross-sectional [43], longitudinal [44], qualitative [31], and mixed-methods [45]) and measures of both food insecurity and academic performance. Although individual studies and narrative reviews highlight associations between food insecurity and academic outcomes [2], [24], [25], [26], [28], [29], [30], a comprehensive synthesis of the available evidence is needed to better understand the breadth, characteristics, and consistency of findings across the literature. Recently, Galseku et al. [46] published the first systematic review examining the association between food insecurity and academic performance among higher education students. However, that review was limited to quantitative cross-sectional and longitudinal studies and did not include qualitative or mixed-methods research. Consequently, it did not capture students’ lived experiences or provide a comprehensive synthesis of the potential mechanisms and pathways linking food insecurity to academic outcomes. In addition, the exclusion of qualitative and mixed-methods studies may have limited the representation of evidence from certain geographic and institutional contexts. A broader review is therefore needed to examine how academic performance has been conceptualized and measured across diverse study designs, identify at-risk populations and settings, and synthesize the proposed mechanisms through which food insecurity may influence academic success. Scoping reviews are particularly well suited for mapping broad and heterogeneous bodies of literature, identifying research trends, and clarifying knowledge gaps [47].

Therefore, the objective of this scoping review was to systematically identify, map, and summarize the existing literature on the relationship between food insecurity and academic performance among postsecondary students worldwide. Specifically, this review sought to describe how food insecurity and academic performance have been measured, summarize reported associations and proposed mechanisms, and identify priorities for future research and intervention efforts.

## Methods

### Protocol and registration

This scoping review was conducted in accordance with the PRISMA extension for Scoping Reviews (PRISMA-ScR) guidelines [48]. A review protocol was developed a priori by the research team to guide the conduct of the review. The protocol was followed throughout the conduct of this scoping review but was not prospectively registered. The conduct and reporting of the review adhered to the PRISMA-ScR checklist to enhance transparency and methodological rigor.

### Eligibility criteria

A detailed summary of the inclusion and exclusion criteria is provided in Table 1. Eligibility criteria were defined a priori using the Population, Concept, Context (PCC) framework recommended for scoping reviews.

**TABLE 1:** Inclusion and exclusion criteria

Criterion	Inclusion	Exclusion
**Population**	University or higher-education students (undergraduate, graduate, or professional)	Primary or secondary school students
**Concept**	Food insecurity, food insufficiency, hunger, access to food	Studies unrelated to food insecurity (e.g., only nutrition quality or dietary intake without insecurity)
**Context/outcome**	Academic performance, academic achievement, GPA, grades, learning outcomes, retention, and graduation	Studies that measure only mental health, physical health, or food security policy without academic outcomes
**Study type**	Quantitative, qualitative, or mixed-methods studies; gray literature (reports, policy documents)	Reviews, commentaries, opinion pieces, theses or dissertations, or media articles without empirical data
**Language**	English only	Non-English publications
**Time frame**	No time restriction	None

Abbreviation: GPA, grade point average.

#### Population

The population of interest included postsecondary students enrolled in universities, colleges, or other higher education institutions, including undergraduate, graduate, and professional students. Studies focusing exclusively on primary or secondary school students were excluded.

#### Concept

The concept of interest was food insecurity, broadly defined to include food insecurity, food insufficiency, hunger, or inadequate access to food. Studies that addressed nutrition-related issues without reference to food insecurity (e.g., dietary quality or nutrient intake alone) were excluded.

#### Context/outcome

The context was academic performance and educational outcomes, including both objective and student-reported academic measures. Eligible outcomes included academic performance, academic achievement, GPA, grades, learning outcomes, academic standing, retention, persistence, and graduation. For qualitative studies, eligible outcomes also included students’ reported experiences of food insecurity affecting their academic functioning, such as difficulty concentrating, attending classes, completing coursework, studying, or maintaining academic performance. Studies that focused solely on mental health, physical health, or food security policies without academic outcomes were excluded.

#### Study characteristics

This scoping review included quantitative, mixed-methods, and qualitative studies that examined the relationship between food insecurity and academic outcomes among postsecondary students. Quantitative studies were required to report measurable academic outcomes, whereas qualitative studies were included if they examined students’ reported academic functioning or perceived academic impacts of food insecurity. In addition to peer-reviewed journal articles, this scoping review included relevant gray literature reports that presented original empirical data on food insecurity and academic outcomes among postsecondary students. Reports were required to describe data sources and analytic methods and were screened using the same eligibility criteria as peer-reviewed studies. Reviews, commentaries, opinion pieces, theses or dissertations, and media articles without original empirical findings were excluded.

#### Language and time frame

Only studies published in English were included. No restrictions were applied regarding publication year.

### Information sources

A comprehensive search was conducted across multiple academic databases, including MEDLINE, Web of Science Core Collection, ERIC, Embase, CINAHL, and PsycINFO. ERIC was included in part because it indexes both peer-reviewed literature and selected grey literature relevant to education research. To further ensure comprehensive coverage of gray literature, additional searches were conducted using Google Scholar (first 100 results), including reports and working papers produced by research institutes, nonprofit evaluation organizations, and professional research societies. In addition, the reference lists of included articles, as well as relevant reviews and key papers, were manually screened to identify additional eligible sources (snowballing). No restrictions were applied regarding publication date. The final search was conducted on 2 November, 2025.

### Search strategy

The search strategy was developed by the research team to identify literature examining the relationship between food insecurity and academic outcomes among postsecondary students. An initial scoping search was conducted in MEDLINE to identify relevant studies and refine terminology related to food insecurity, academic performance, and student populations. Keywords and indexing terms derived from these records informed the development of the final search strategy.

### Key search concepts and operationalization

The search strategy was structured around 3 core concepts. Food insecurity as the primary exposure, academic performance as the context or outcome, and students in higher or tertiary education as the population of interest. Each concept was operationalized using a comprehensive set of related terms, which were combined using Boolean operators. The 3 concepts were linked using AND to ensure retrieval of studies addressing all elements of the research question.

### Use of controlled vocabulary and free-text terms

Controlled vocabulary terms were incorporated where available to enhance search precision. In MEDLINE, Medical Subject Headings such as “Food Insecurity,” “academic performance,” “academic achievement,” and “Students” were used. These terms were supplemented with free-text keywords searched in titles and abstracts to increase sensitivity and capture variations in terminology across disciplines. The complete search strategy for MEDLINE, including all controlled vocabulary terms, free-text terms, and Boolean operators, is provided in Supplemental Table 1. This strategy was adapted for use in other databases by modifying subject headings and search syntax as appropriate.

### Selection of sources of evidence

The selection of evidence sources followed a 2-stage screening process. All records identified through the database searches were imported into the review management system, and duplicates were removed before screening. In the first stage, titles and abstracts were screened to assess potential relevance to the review objectives. Records that clearly did not meet the inclusion criteria were excluded at this stage. In the second stage, full-text articles of all potentially relevant records were retrieved and assessed for eligibility. Full-text screening focused on confirming alignment with the PCC criteria defined a priori.

Title and abstract screening was conducted independently and in duplicate by 2 reviewers (EA and AM). Full-text screening was also performed independently by the same reviewers. Any discrepancies at the screening stage were resolved through discussion and consensus. When agreement could not be reached, a third reviewer (MS) was consulted to make the final determination. Screening and study management were supported using Rayyan systematic review software, which facilitated independent screening, record management, and documentation of reasons for exclusion at the full-text stage.

### Data charting process

Data charting was conducted using a structured data charting form developed by the review team to systematically extract relevant information from the included evidence sources. To ensure appropriate capture of study-specific characteristics and findings, separate but aligned charting fields were created for quantitative and qualitative studies. The data charting form was piloted on a subset of included studies representing quantitative, qualitative, and mixed-methods designs. Minor refinements were made after the pilot phase to improve clarity, consistency, and completeness of the charting fields.

Data charting was completed independently and in duplicate by 2 reviewers (EA and AM). Extracted data were compared, and any inconsistencies were resolved through discussion and consensus. When discrepancies could not be resolved, a third reviewer (MS) was consulted. The charting form was refined iteratively throughout the process to accommodate emerging study characteristics and themes, with all modifications agreed upon by the review team to maintain methodological consistency.

### Data items

For quantitative studies, the following data elements were extracted: author(s) and year of publication, country and study setting, sample size, population characteristics, food security measurement tool, academic performance measure(s), and key findings related to the association between food insecurity and academic outcomes. For qualitative studies, extracted data included author(s) and year of publication, country and study setting, sample size, population characteristics, qualitative study design, analytic approach, and key qualitative themes related to food insecurity and academic performance. For mixed-methods studies, relevant quantitative and qualitative data items were extracted using the corresponding charting fields.

### Critical appraisal of individual evidence sources

A formal critical appraisal of individual evidence sources was not conducted in this scoping review. This decision aligns with the primary review purpose to map and synthesize the breadth, characteristics, and scope of the existing global literature on the relationship between food insecurity and academic performance among postsecondary students. The absence of formal appraisal allows for a comprehensive overview of the literature and facilitates the identification of knowledge gaps to inform future research.

### Synthesis of results

The extracted data were synthesized using a descriptive and narrative approach, consistent with the scoping review objectives. Quantitative findings were summarized using descriptive statistics, including the number of studies, publication years, countries and regions, study designs, populations, food insecurity measurement tools, and academic outcome measures. Qualitative findings were synthesized using a narrative thematic approach, whereby key themes related to food insecurity and academic performance were identified and summarized across studies. For mixed-methods studies, quantitative and qualitative findings were synthesized and reported within their respective components.

The results were organized to map the existing evidence base, highlighting patterns, similarities, and gaps in the literature regarding the relationship between food insecurity and academic outcomes among postsecondary students. Findings were presented using a combination of summary tables, figures, and narrative descriptions to facilitate comparison across studies and enhance interpretability. No meta-analysis or quantitative pooling of results was undertaken, as the purpose of this review was to describe the scope and nature of the available evidence rather than to estimate effect sizes.

## Results

### Selection of sources of evidence

The database searches yielded a total of 3078 records. After removal of 649 duplicates, 2429 records remained for title and abstract screening. Of these, 2299 records were excluded at the title and abstract stage because they did not meet the inclusion criteria. A total of 130 full-text articles were retrieved and assessed for eligibility. After full-text review, 45 articles were excluded for the following reasons: population not relevant (*n* = 5), concept not relevant (*n* = 11), study design not eligible (*n* = 3), ineligible publication type (*n* = 21), and outcomes not relevant (*n* = 5). In total, 86 sources of evidence met the inclusion criteria and were included in the scoping review, including 84 peer-reviewed studies and 2 gray literature sources. The study selection process is summarized in the PRISMA-ScR flow diagram (Figure 1).

**FIGURE 1:**
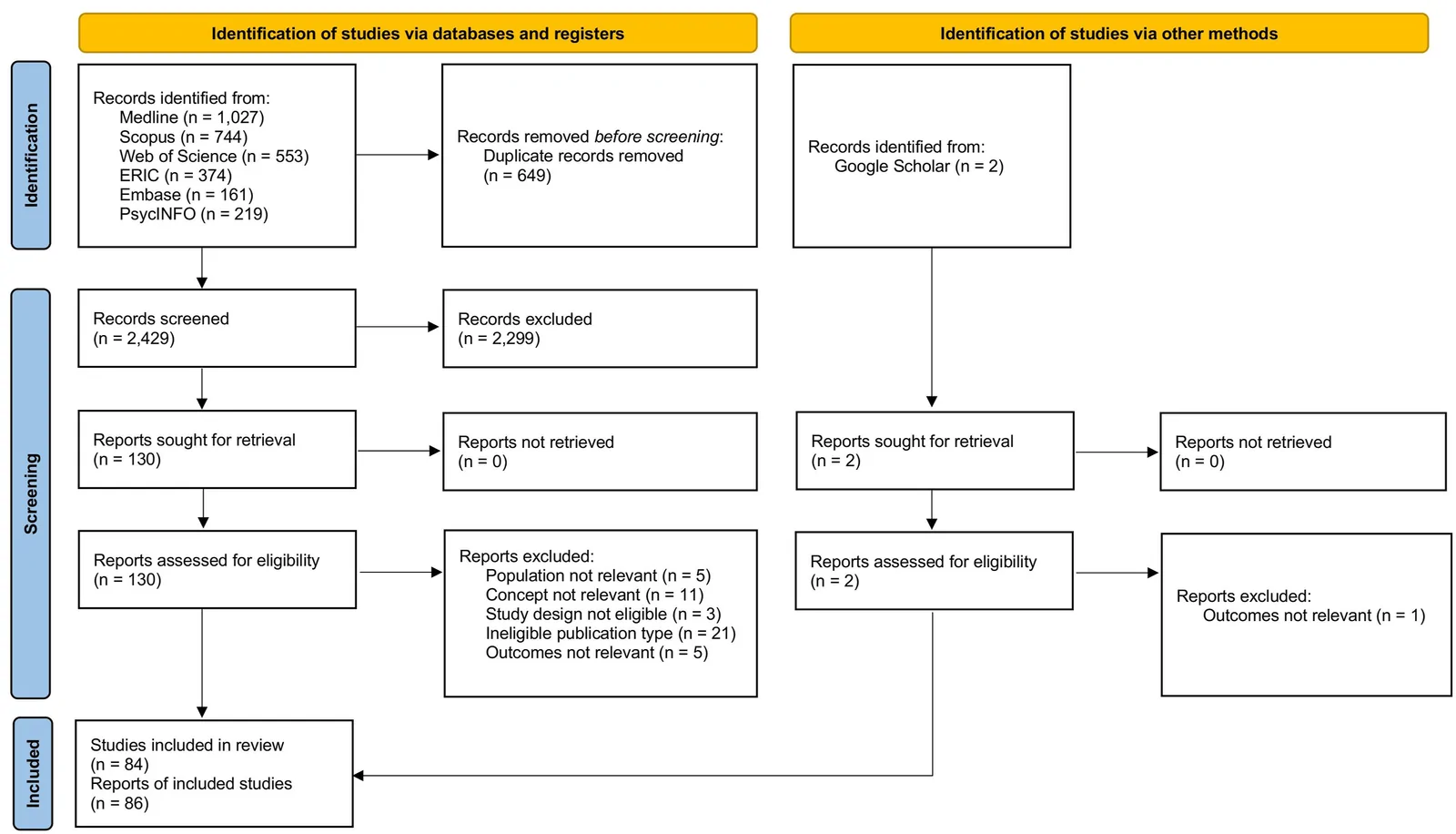
PRISMA flow diagram.

### Characteristics of included sources

The characteristics of the included studies are summarized in Tables 2, 3, and 4 [49], [50], [51], [52], [53], [54], [55], [56], [57], [58], [59], [60], [61], [62], [63], [64], [65], [66], [67], [68], [69], [70], [71], [72], [73], [74], [75], [76], [77], [78], [79], [80], [81], [82], [83], [84], [85], [86], [87], [88], [89], [90], [91], [92], [93], [94], [95], [96], [97], [98], [99], [100], [101], [102], [103], [104], [105], [106], [107], [108], [109], [110], [111], [112], [113]. The 86 included articles represented 84 unique study samples. The majority of studies employed quantitative designs (*n* = 67), including 59 cross-sectional and 8 longitudinal studies. Qualitative approaches were used in 11 studies, whereas 8 studies employed a mixed-methods design. The included studies were published between 2015 and 2025, with a noticeable increase in publications over time. Few studies were published before 2018, followed by a steady rise from 2019 onward. The highest number of publications occurred in 2023 (*n* = 13), with continued high publication volume in 2024 (*n* = 12) and 2025 (*n* = 12). Most studies were conducted in the United States (*n* = 69) (Figure 2), with a smaller number of studies from Canada (*n* = 5) and Malaysia (*n* = 4). Single studies were conducted in Australia, Germany, Hungary, Iceland, Jordan, Mexico, Saudi Arabia, and South Africa (*n* = 1 each). No eligible studies were identified from Sub-Saharan Africa, East Asia, or South America beyond Mexico.

**TABLE 2:** Characteristics of the included quantitative studies

Author (y)	Country/setting		Sample size	Population studied	FS measure	AP measure	Key findings
**Cross-sectional**							
**Adkisson et al. (2023)**[49]		United States (single university in the Southeastern United States)	169 married student households	Married undergraduate and graduate university students (household-level analysis; with and without children)	USDA Household Food Security Survey (18-item HFSS for households with children; 10-item AFSS for households without children)	• Self-reported perceived academic performance (pandemic vs. prepandemic)	Food-insecure married student households, especially those with children, reported significantly worse academic performance during COVID-19
**Ahmad et al. (2021)**[50]		Malaysia (Universiti Putra Malaysia, public research university)	663 undergraduate students	Undergraduate university students (second- and third- year students)	USDA-FSSM, 6-item version	• Self-reported CGPA • CGPA categorized as ≥3.7 vs. <3.7 (UPM grading system)	Food-insecure university students were significantly less likely to have a CGPA ≥3.7 and had higher odds of stress, anxiety, and depression than food-secure students
**Ahmad et al. (2022)**[51]		Malaysia (1 public research university: Universiti Putra Malaysia)	663 bachelor’s degree students	Undergraduate university students (second- and third-year students)	United States AFSSM (US-FSSM), 10-item version	• Self-reported CGPA • CGPA categorized as ≥3.75 vs. <3.75 based on university grading system	Food insecurity was significantly associated with poorer academic performance, and this relationship was partially mediated by higher levels of anxiety and depression among university students
**Ahmed et al. (2023)**[52]		United States (New York City; 3 public CUNY colleges—2 community colleges and one 4-y college)	1989 undergraduate students	Undergraduate students at public colleges (community college and 4-y university students)	USDA 10-item AFSSM, past 3 mo	• Self-reported GPA • Self-reported effect of food insecurity on academic performance (composite scale assessing concentration, studying, completing assignments, class attendance, course withdrawal/failure)	Across prepandemic and during-pandemic periods, food insecurity was significantly associated with worse academic performance, with students reporting greater difficulty concentrating, studying, completing coursework, attending class, and higher likelihood of course withdrawal or failure
**Alsharafat et al. (2024)**[36]		Jordan (Jerash University; public university)	126 postgraduate students	Postgraduate university students (Master’s and doctoral students)	Modified USDA Adult HFSSM; adapted to 8 items	• GPA obtained from the university Department of Registration	Food insecurity was inversely associated with academic performance, with postgraduate students experiencing marginal to very low food security demonstrating significantly lower GPAs than food-secure students
**Beam & Johnson (2023)**[28]		United States (one 4-y public university in the Midwestern United States)	314 undergraduate students (from a random sample of 2700 invited students)	Undergraduate students at a 4-y public university (college/university level)	USDA AFSSM (10-item), past 12 mo	• Self-reported CGPA • Self-reported academic outcomes (failing a course, withdrawing from a course, taking an incomplete, class attendance, academic engagement)	Students experiencing food insecurity had significantly lower GPAs and were more likely to fail or withdraw from courses and take incomplete grades vs. food-secure students
**Becerra et al. (2023)**[53]		United States (mid-sized 4-y public university; Hispanic-Serving and Minority-Serving Institution in California)	223 college students	Undergraduate and graduate college students at a 4-y public university	USDA Food Security Questionnaire	• CGPA • Self-reported indicator of academic disruption	Food insecurity was significantly associated with worsened academic performance during the COVID-19 pandemic, particularly among racial/ethnic minority female students, although it was not significantly associated with CGPA in the full sample
**Betancourt-Núñez et al. (2024)**[38]		Mexico (public university in western Mexico; Universidad de Guadalajara)	466 university students	Undergraduate and postgraduate university students	ELCSA; 8–15 items depending on household composition	• Self-reported AG; cumulative average on a 0–100 scale • Self-reported indicators of academic performance (overall progress, class attendance, attention in class, understanding of concepts, perceived academic future)	Food insecurity was not significantly associated with cumulative academic grading but was significantly associated with poorer self-perceived academic progress, class attendance, and perceived ability to complete studies, even after accounting for mental health and sleep quality
**Brandhorst et al. (2023)**[54]		United States (Michigan State University; large Big Ten public research university)	1408 students with complete food security data (654 undergraduates; 754 graduate students)	Undergraduate and graduate university students (analyzed separately)	USDA 6-item short-form AFSSM (reference period: academic year)	• Self-reported GPA • Self-reported academic functioning indicators (concentration, energy level, overall well-being)	Higher food security was associated with higher GPAs and better academic-related functioning, with undergraduates experiencing food insecurity at higher rates and showing a stronger clustering of lower GPAs than graduate students
**Brescia and Cuite (2022)**[55]		United States (Rutgers University–New Brunswick; large public research university in the Northeastern United States)	6823 undergraduate students completed the survey; 4491 undergraduates included in GPA-based analyses	Undergraduate students at a 4-y public research university	USDA 10-item AFSSM (categorizing high, marginal, low, and very low food security)	• CGPA obtained from institutional records	Marginally food-secure students had significantly lower GPAs than highly food-secure students and did not differ significantly from students with low food security, suggesting that marginal food security is academically more like food insecurity than food security
**Brownfield et al. (2023)**[35]		Australia (public university in Melbourne; Swinburne University of Technology)	664 undergraduate students (usable cases with linked WAM)	Undergraduate university students (ages 18–25 y)	Adapted USDA 6-item CPS-FSS, assessed across students’ studies	• WAM obtained from university records (0–100 scale)	Food insecurity was significantly associated with lower WAM, and this relationship was partially mediated by higher psychological distress among university students
**Budnick et al. (2025)**[56]		United States (large public university in Upstate New York)	292 validated undergraduate responses across 3 survey waves (fall 2022, spring 2023, fall 2023)	Undergraduate college students (ages 18–26 y)	Self-constructed food insecurity score assessing financial worry, frequency of inadequate food access, and eligibility for subsidized lunch programs	• Self-reported GPA from the previous academic semester	Higher food insecurity was significantly associated with lower GPA, as well as higher stress, anxiety, and depression among college students.
**Camelo and Elliott (2019)**[57]		United States (1 midsize public land-grant university in the western United States)	3245 undergraduate students	Undergraduate students at a public 4-y research university	Modified USDA 6-item HFSSM, adapted for college students and analyzed as a continuous frequency score (mean of 5 items reflecting frequency of food insecurity)	• CGPA obtained from institutional records (end of Spring 2016 semester)	Greater frequency of food insecurity was significantly associated with lower GPA, and food insecurity partially mediated the relationships between race/ethnicity, socioeconomic status (e.g., Pell eligibility, first-generation status), and academic achievement
**Cockerham et al. (2021)**[58]		United States (Sam Houston State University, Texas; public university nursing program)	55 undergraduate nursing students (from 293 enrolled; 18.8% response rate)	Undergraduate baccalaureate nursing students (junior and senior level)	USDA 6-item short-form HFSSM	• Self-reported CGPA (categorized as ≥3.0 vs. <3.0)	Food-insecure nursing students had significantly lower GPAs than food-secure students, and food insecurity was more prevalent among first-generation students
**DeBate et al. (2021)**[59]		United States (large urban public research university in the Southeastern United States)	1743 college students	Undergraduate and graduate college students (majority undergraduate; full- and part-time)	USDA 6-item short-form FSSM (self-administered; categorized as high/marginal, low, very low food security)	• Self-reported CGPA • Self-reported indicators of academic challenges (academics, career planning, procrastination, faculty engagement)	Students experiencing any level of food insecurity had significantly lower GPAs and were more likely to report academic challenges related to coursework, procrastination, career planning, and faculty interactions vs. food-secure students
**El Zein et al. (2019)**[60]		United States (8 public universities across multiple regions of the United States)	855 first-year college students	First-year undergraduate college students	USDA AFSSM; 10-item, past 12 mo; categorized into high/marginal vs. low/very low food security	• Self-reported CGPA (categorized ranges; GPA <3.0 vs. ≥3.0 used in regression analyses)	Food-insecure first-year college students had nearly twice the odds of having a GPA of <3.0 vs. food-secure students, even after adjusting for sociodemographic and meal-plan factors
**Berger et al. (2024)**[29]		United States CUNY (large public urban university system)	2282 college students (population-representative weighted sample)	Undergraduate and graduate students across associate, bachelor’s, and graduate degree programs	4 items adapted from the USDA 6-item Short Form HFSSM (assessing food insecurity in the prior 2 wk; categorized as food secure, moderate food insecurity, high food insecurity)	• Self-reported educational disruptions due to COVID-19 (decreased ability to do schoolwork, dropping or withdrawing from classes, the expected delays or uncertainty in graduation)	Moderate and high food insecurity were significantly associated with decreased ability to do schoolwork, higher likelihood of dropping or withdrawing from classes, and anticipated delays in graduation, with anxiety and depression partially mediating these relationships
**Faidi et al. (2023)**[61]		Malaysia (public universities nationwide; multicampus sample)	300 undergraduate university students	Undergraduate students at public universities (ages 18–25 y)	USDA AFSSM; 10-item; categorized as high, marginal, low, very low food security	• Self-reported CGPA, categorized by institutional grading criteria	Food insecurity was significantly associated with lower CGPA among Malaysian university students during the COVID-19 pandemic
**Farahbakhsh et al. (2017)**[62]		Canada (University of Alberta; large public research university in Edmonton, Alberta)	58 university students who were clients of the campus food bank	Undergraduate and graduate university students receiving emergency food assistance	USDA AFSSM; 10-item; past 30 d; categorized as food secure, moderately food insecure, severely food insecure using Health Canada scoring	• Self-reported adverse academic experiences due to lack of money for food (e.g., inability to concentrate in class or during examinations, inability to study, incomplete assignments, failing or withdrawing from a course)	Students with severe food insecurity were significantly more likely to report adverse academic experiences, particularly difficulty concentrating in class or during examinations and failing or withdrawing from a course, vs. students with nonsevere food insecurity
**Fowler and Hesson (2025)**[34]		Canada (Single Atlantic Canadian university, Memorial University of Newfoundland, St. John’s, Newfoundland and Labrador)	1694 university students	Undergraduate and graduate university students. Includes domestic and international students, with diverse racial and ethnic backgrounds	USDA Economic Research Service 6-Item Food Security Short Form	• Self-reported approximate GPA, categorized as A, B, C, D, or F	Food insecurity was prevalent (≈47%) and was significantly associated with lower GPA among university students. Food-insecure students also reported greater psychological distress and lower social support, with food insecurity independently linked to poorer academic performance
**Gilster et al. (2023)**[63]		United States (public Midwestern university; School of Social Work)	125 undergraduate and graduate social work students (analytic sample)	Upper-level undergraduate (BSW) and graduate (MSW) university students	USDA 6-item short-form HFSSM (past 12 mo; categorized as food secure, low food security, very low food security)	• Self-reported CGPA; 0–4.33 scale	Very low food security was significantly associated with lower GPA, even after controlling for housing insecurity, degree program (BSW vs. MSW), and demographic covariates
**Hagedorn and Olfert (2018)**[64]		United States (large public land-grant university in Central Appalachia; West Virginia University)	692 undergraduate and graduate students (final analytic sample)	Undergraduate and graduate students at a public research university	USDA AFSSM (10-item; past 12 mo; categorized as high, marginal, low, and very low food security)	• Self-reported CGPA • APS: self-reported class attendance, attention span, understanding of course material, and progress toward graduating on time	Food-insecure students had significantly lower GPAs and lower academic progress scores, indicating poorer attendance, concentration, comprehension, and progression toward graduation than food-secure students
**Hagedorn et al. (2019)**[65]		United States (10 public 4-y universities across the Appalachian and Southeastern regions; Mississippi, North Carolina, Tennessee, West Virginia)	13,642 students with complete food security data	Undergraduate and graduate students enrolled at public 4-y universities	USDA AFSSM (AFSS; 10-item; past 12 mo; categorized as high/marginal vs. low/very low food security)	• GPA; self-reported • APS: perceived class attendance, attention span, comprehension of course material, and progress toward on-time graduation	Food-insecure students had significantly lower GPAs and poorer academic progress scores, indicating worse attendance, concentration, comprehension, and progression toward graduation than food-secure students
**Haro-Contreras et al. (2025)**[66]		United States (University of California system; 9 UC campuses in California)	• 592 undocumented students • 634 United States citizens with ≥1 undocumented parent • 635 United States citizens with lawfully present parents.	Undergraduate university students (undocumented students, United States citizens with undocumented parents, and United States citizens with lawfully present parents)	Adapted USDA HFSSM (short form; 4–5 items assessing food affordability and hunger over the past 12 mo; categorized as food secure vs. low/very low food security)	• Self-reported CGPA (0.0–4.0 scale) • Self-reported decline in academic achievement during the study period	Food insecurity was significantly associated with a decline in academic achievement (lower GPA), even after controlling for demographic factors, mental health, and family economic hardship among Latinx immigration-impacted students
**Harville et al. (2023)**[43]		United States (University of Florida; large public southeastern research university)	1087 undergraduate students (randomized sample; 21.7% response rate)	Undergraduate college students (full-time and part-time)	USDA 6-item short-form FSSM (past 12 mo; dichotomized as food secure vs. food insecure)	• Self-reported CGPA	Food-insecure students had significantly lower GPAs than food-secure students, with food insecurity disproportionately affecting non-White, first-generation, and financial-aid-receiving students
**Haskett et al. (2021)**[67]		United States (large public university in the Southeastern United States; North Carolina State University)	1330 undergraduate and graduate students (from a random sample of 7000 invited; 28% response	Undergraduate and graduate university students (primarily full-time)	USDA 10-item HFSSM/Individual FSSM (30-d timeframe; categorized as food secure, marginally food secure, low food security, very low food security; often dichotomized as food secure vs. food insecure)	• Self-reported CGPA (categorized ranges; collapsed to <2.5 vs. ≥2.5 in analyses)	Food insecurity was significantly associated with lower GPA, and this relationship was partially mediated by poorer psychological well-being and fewer hours of sleep, whereas homelessness was not significantly associated with GPA
**Hege et al. (2021)**[68]		United States (University of Kentucky; large public land-grant university in Kentucky)	1632 students who fully completed the survey (from 1854 survey starters)	Undergraduate and graduate university students (majority undergraduate; full-time and part-time)	USDA 6-item short-form AFSSM (12-mo recall; categorized as food secure vs. food insecure)	• CGPA (self-reported; analyzed categorically, e.g., GPA <2.5 vs. ≥2.5) • Self-reported academic disruption indicators (difficulty concentrating on studies, not purchasing required course materials, suspending or dropping studies due to finances)	Food-insecure students were significantly more likely to have lower GPAs and to experience academic disruptions, including difficulty concentrating, not purchasing required course materials, and suspending or dropping studies because of financial constraints
**Tin et al. (2022)**[69]		United States (multicampus community college district in California; urban, suburban, and rural campuses)	238 community college students (from a stratified random sample of 5000 invited)	Community college students (associate degree–seeking; adult students aged 18+)	USDA AFSSM; 10-item; past 12 mo; categorized as high, marginal, low, and very low food security; also dichotomized as food secure vs. food insecure	• Self-reported CGPA (categorized ranges; emphasis on GPA ≥3.5 vs. lower GPA groups)	Students who reported being food secure were significantly more likely to have a GPA of ≥3.5, whereas food-insecure students were overrepresented in lower GPA categories
**Kahtan and Hanbazaza (2024)**[37]		Saudi Arabia (King Abdulaziz University, Jeddah; large public university)	338 university students with complete food security data (from 421 respondents)	Undergraduate and postgraduate university students	FIES; 8-item scale; past 30 d; categorized as food secure, mild, moderate, or severe food insecurity	• Self-reported CGPA (categorized using the university grading scale) • APS: self-reported class attendance, attention span, comprehension of course material, and progress toward timely graduation	Food insecurity was not significantly associated with CGPA or academic progress scores, although it was significantly associated with poorer mental health, poorer general health, and lower household income
**Kötzsche et al. (2025)**[70]		Germany (Justus Liebig University Giessen; public university)	562 university students (final analytic sample)	Undergraduate and postgraduate university students	FIES; 8-item FAO scale; past 12 mo; categorized as food secure vs. food insecure	• Self-reported impact of the COVID-19 pandemic on academic and/or research progression (5-point Likert Scale ranging from very negative to very positive)	Food-insecure students were significantly more likely to report a negative impact of the COVID-19 pandemic on their academic and/or research progression vs. food-secure students
**Soria and Smith (2024)**[40]		United States (113 community colleges across multiple states)	3352 community college student-veterans (from a broader survey of 88,733 community college students)	Community college students (undergraduate student veterans)	USDA 18-item HFSSM (30-d reference period; categorized as high/marginal, low, and very low food security; dichotomized for food insecurity)	• Self-reported GPA (current term GPA)	Community college student veterans who experienced food insecurity (as part of basic needs insecurity) reported significantly lower self-reported GPAs than student veterans who did not experience basic needs insecurity
**C. W. Leung et al. (2021)**[71].		United States (University of Michigan; large public Midwestern research university)	793 college students (analytic sample from 851 respondents; 43% response rate)	Undergraduate and graduate college students at a 4-y public university	USDA 10-item AFSSM (past 12 mo; summed score 0–10; food insecurity defined as ≥3 affirmative responses)	• CGPA obtained from institutional records (self-reported only for students who did not consent to record linkage)	Students experiencing food insecurity, especially those facing multiple basic-needs insecurities, had significantly lower GPAs than fully secure students, even after adjusting for sociodemographic characteristics
**Lofgren (2025)**[72]		United States (large urban public university in the Southeastern United States; Georgia State University)	520 university students (final analytic sample from a random sample of 7940 invited)	Undergraduate and graduate university students	Single-item indicator adapted from USDA food security constructs: Self-reported worry about having enough food to eat (used as a proxy for moderate food insecurity)	• Self-reported negative academic outcomes resulting from attendance policies (lowered grade, course failure, or course withdrawal despite otherwise passing performance)	Food insecurity and broader poverty-related ill-being were significant predictors of negative academic outcomes, such as grade reductions, course failure, or withdrawal, through the enforcement of punitive attendance policies
**Marmolejo et al. (2024)**[73]		United States (75 colleges and universities nationwide; ACHA–National College Health Assessment III)	48,103 college students (final analytic sample from 75 United States institutions)	Undergraduate and graduate college students (majority undergraduate; full-time and part-time)	USDA 6-item short-form HFSSM (self-administered; categorized as high/marginal, low, and very low food security; low + very low combined as food insecure for regression analyses)	• Self-reported CGPA (converted from letter to grade categories to continuous and binary measures, e.g., A− or higher vs. lower than A−)	Low and very low food security were significantly associated with lower GPA, and this relationship persisted even after accounting for psychological distress, loneliness, psychological well-being, and sociodemographic covariates
**Maroto et al. (2015)**[74]		United States (2 community colleges in Maryland: 1 urban, 1 suburban)	301 community college students	Community college students (associate degree–seeking; ages 18+)	USDA 10-item AFSSM; past 12 mo; categorized as high/marginal vs. low/very low food security using USDA scoring	• Self-reported CGPA (reported in GPA ranges, e.g., 2.0–2.49 vs. 3.5–4.0)	Food-insecure community college students were significantly less likely to have high GPAs (3.5–4.0) and more likely to fall into lower GPA categories (2.0–2.49) than food-secure students
**Marshall et al. (2021)**[75]		United States (University of Iowa; public Midwestern dental school)	81 dental students (24.7% response rate from 328 invited)	Professional doctoral students (dental students; years 1–4)	USDA 10-item AFSSM (categorized as food secure vs. food insecure, with marginal, low, and very low combined as food insecure)	• Self-reported food- or hunger-related academic performance difficulties (e.g., difficulty studying, perceived academic performance problems) • GPA reported in categories (3.5–4.0 vs. <3.5)	Food insecurity was significantly associated with food- or hunger-related academic performance difficulties and stress, although it was not directly associated with GPA in this dental student population
**Martinez et al. (2020)**[25]		United States (University of California system; all 10 UC campuses statewide)	8705 college students with complete food security data (7999 with complete GPA data)	Undergraduate and graduate students enrolled in a public university system	USDA 6-item short-form FSSM (past 12 mo; categorized as food secure [0–1] vs. food insecure [2–6 affirmative responses])	• Self-reported CGPA (letter grades converted to numeric scale: D/F = 1 to A = 4)	Food insecurity was significantly associated with lower GPA both directly and indirectly through poorer mental health, indicating that mental health partially mediated the relationship between food insecurity and academic performance
**Martinez et al. (2018)**[76]		United States (University of California system; 10 public research universities statewide in California)	8705 undergraduate and graduate students with complete food security data (from 10 UC campuses)	Undergraduate and graduate university students enrolled in a public university system	USDA 6-item short-form FSSM (past 12 mo; categorized as food secure [0–1], low food security [2], [3], [4], very low food security [5], [6]; low + very low combined as food insecure)	• Self-reported CGPA (reported in letter-grade categories: A, B, C, D/F) • Self-reported academic consequences related to food insecurity (e.g., difficulty concentrating because of hunger; suspending studies because of financial hardship)	Food-insecure students were significantly more likely to report lower CGPA and academic difficulties such as trouble concentrating because of hunger and suspending studies because of financial hardship vs. food-secure students
**McArthur et al. (2018)**[77]		United States (Appalachian State University, North Carolina; public university in the Appalachian region)	1093 college students (final analytic sample from 6000 invited)	Sophomore through graduate university students (freshmen excluded)	USDA 10-item AFSSM (past 12 mo; categorized as high/marginal vs. low/very low food security using USDA scoring)	• Self-reported CGPA • APS: self-reported class attendance, attention span, understanding of course material, and overall academic progress (graduating on time)	Greater food insecurity was significantly associated with lower GPA and poorer academic progress, including worse class attendance, attention span, understanding of course material, and perceived progress toward graduation
**Mechler et al. (2024)**[78]		United States (University of New Mexico; large public urban R1, Minority-Serving and Hispanic-Serving Institution in the Southwestern United States)	2654 students (final analytic sample; 22.1% response rate from 12,000 invited)	Undergraduate, graduate, and professional students (including law and medical students)	USDA 10-item AFSSM [past 30 d; categorized as food secure (high/marginal) vs. food insecure (low/very low)]	• Institutional records of academic persistence (enrollment in fall 2020 and spring 2021 or graduation) • Institutional records of academic disruption (number of courses failed or withdrawn; credit hours lost)	Food-insecure students were significantly more likely to fail or withdraw from courses and to stop persisting in enrollment, with very low food security associated with the highest odds of attrition even after controlling for demographic factors
**Morris et al. (2016)**[79]		United States (4 public universities in Illinois: Eastern Illinois University, Northern Illinois University, Southern Illinois University, and Western Illinois University)	1882 undergraduate students	Undergraduate university students (juniors and seniors overrepresented)	USDA HFSSM (10-item; past 9 mo; categorized as high, marginal, low, and very low food security; low + very low classified as food insecure)	• Self-reported CGPA, reported in categorical ranges (0.00–1.99, 2.00–2.99, 3.00–3.99, 4.00)	Food insecurity was significantly associated with lower GPA, with students in lower GPA categories exhibiting significantly higher rates of low and very low food security vs. students with higher GPAs
**Moya et al. (2025)**[80]		United States (University of Texas at El Paso; large public R1 Hispanic-Serving Institution in the United States–Mexico border region)	2020 university students completed the survey (population-weighted analytic sample = 1729)	Undergraduate, master’s, and doctoral university students (majority undergraduate; predominantly Hispanic)	USDA 6-item short-form FSSM (past 12 mo; categorized as high/marginal, low, and very low food security)	• Self-reported difficulty concentrating on academic work due to concerns about food or other necessities • Self-reported consideration of delaying degree completion • Self-reported actual degree delay due to financial constraints (Note: GPA was not measured)	Low and very low food security were strongly associated with poorer academic concentration and significantly increased likelihood of thinking about or experiencing degree delays among students at a Hispanic-Serving Institution
**Payne-Sturges et al. (2018)**[81]		United States (large public mid-Atlantic university; University of Maryland, College Park)	237 undergraduate students	Undergraduate university students	USDA 18-item HFSSM; past 12 mo; categorized as food secure, at risk/marginal food security, and food insecure following USDA protocol	• Self-reported CGPA • Self-reported academic disruption related to depression (e.g., depression symptoms impacting schoolwork)	Food-insecure and at-risk students reported significantly poorer academic functioning related to depression symptoms, although no statistically significant difference in GPA was observed by food security status
**Phillips et al. (2018)**[82]		United States (large public, 4-y Midwestern university)	508 undergraduate students (GPA analyses conducted on a subsample of 340 students who consented to record access)	Undergraduate university students	USDA 6-item short-form FSSM (past 12 mo; categorized as food secure/marginal, low food security, and very low food security following USDA guidelines)	• CGPA from official university records • Self-reported indicators of academic disruption (neglecting academics, reducing course load, considering dropping out due to financial constraints)	Food insecurity was significantly associated with lower CGPA and substantially higher odds of academic disruption, including neglecting coursework, reducing course load, and considering dropping out of college
**Rafferty et al. (2023)**[39]		United States (private university in the Midwestern United States)	253 undergraduate and graduate students	Undergraduate and graduate college students	USDA 10-item AFSSM (retrospective pretest–posttest design comparing prepandemic vs. during-pandemic food security) Using Qualtrics	• CGPA (Spring 2020; self-reported) • Self-reported indicator of academic disruption (pandemic-related academic difficulties)	Worsening food security during the COVID-19 pandemic was significantly associated with lower spring 2020 GPA and increased academic difficulties among college students
**Repella et al. (2024)**[83]		Iceland (3 universities: University of Iceland, University of Akureyri, and Reykjavik University)	A total of 924 university students completed the survey; 727 were included in food security analyses due to complete FIES data	Undergraduate and postgraduate university students	FIES; 8-item FAO scale; past 12 mo; categorized as food secure vs. food insecure using modified USDA and FAO scoring approaches	• Self-reported change in academic performance during the COVID-19 pandemic (categorized as negative, positive, or not affected)	Food-insecure students were significantly more likely to report negative changes in academic performance during the COVID-19 pandemic vs. food-secure students
**Ryan et al. (2022)**[84]		United States (private, urban university in New York City; New York University)	257 undergraduate students (final analytic sample)	Undergraduate university students	USDA 6-item United States HFSSM [past 12 o; categorized as food secure (high/marginal) vs. food insecure (low/very low)]	• Self-reported CGPA (dichotomized as <3.0 vs. ≥3.0)	Food insecurity was not significantly associated with CGPA among undergraduate students
**Sabi et al. (2020)**[26]		South Africa (University of KwaZulu-Natal; public university with multiple campuses)	456 university students (91.2% response rate from 500 invited)	Undergraduate and postgraduate university students	9-item measure related to the HFIAS, combined with a 1-item self-reported measure of eating habits (meal frequency under normal circumstances)	• Self-reported indicators of academic disruption (reduced concentration/effectiveness, missed classes due to hunger)	Food insecurity was associated with reduced concentration and effectiveness as a student and increased likelihood of missing classes because of hunger
**Sanborn et al. (2025)**[85]		United States (CUNY; large urban public university system)	3160 matriculated students (population-representative weighted sample)	Undergraduate and graduate university students (community college and senior college students)	USDA 6-item United States HFSSM [past 12 mo; categorized as food secure (high/marginal) vs. food insecure (low/very low)]	• Academic attrition defined as college withdrawal or CGPA meeting criteria for academic probation • GPA thresholds derived from official institutional academic standards and validated against administrative records	Food insecurity was significantly associated with a higher likelihood of academic attrition, and students experiencing both food insecurity and housing instability had more than twice the risk of attrition vs. students who were food and housing secure
**Silva et al. (2017)**[86]		United States (University of Massachusetts Boston; large public urban university)	390 university students	Undergraduate and graduate university students	Self-developed food insecurity items informed by USDA definitions, including frequency of worrying about food, skipping meals, inability to eat balanced meals, and not eating for 1–2 d because of lack of money (severe food insecurity operationalized using the USDA definition of very low food security)	• Self-reported impact of food insecurity on class attendance • Self-reported impact of food insecurity on class performance • Self-reported course failure and course withdrawal due to food or housing issues	Food insecurity was significantly associated with poorer class attendance and performance, and students with severe food insecurity were substantially more likely to fail courses or withdraw from classes
**Singh et al. (2024)**[41]		United States (University of California public university system; 9 UC campuses)	1170 undergraduate students (final analytic sample)	Undergraduate university students	USDA 6-item short-form FSSM [most recent academic term; categorized as food secure (0–1) vs. food insecure (2–6)]	• CGPA obtained from official institutional records (scale 1.00–4.00)	Food insecurity was significantly associated with lower CGPA, and students at the intersection of historically racialized status and low socioeconomic position had the lowest GPAs vs. traditional students
**Spaid et al. (2021)**[87]		United States (community colleges on the East Coast of the United States)	858 community college students (combined sample across 3 studies conducted between 2017 and 2020)	Community college students (undergraduate level)	USDA HFSSM (10-item adult module; past 12 mo; categorized as high, marginal, low, and very low food security)	• Self-reported GPA • Self-reported concentration levels • Self-reported energy levels	Food insecurity was significantly associated with lower GPA, poorer concentration, and lower energy levels, although the strength and significance of these relationships varied across the 3 included studies
**Elias et al. (2023)**[88]		Malaysia (International Islamic University Malaysia, Kuantan campus)	383 undergraduate students	Undergraduate university students (health-related programs)	Radimer–Cornell Food Insecurity Scale [8-item scale; categorized as food secure (0–4) vs. food insecure (5–8)]	• Self-reported CGPA, reported in categorical ranges	Food insecurity was significantly associated with lower academic performance, with students reporting lower CGPA more likely to experience food insecurity
**Taylor et al. (2019)**[89]		United States (University of Memphis; multicampus public university in a high-poverty metropolitan area)	524 undergraduate students	Undergraduate university students	6-Item Food Security Scale (USDA short form), embedded within a 44-item survey assessing prior experiences of food insecurity (categorized as having experienced food insecurity vs. never experienced food insecurity)	• Self-reported CGPA • Self-reported indicators of academic disruption (questioning commitment to continuing college education; questioning ability to continue college education)	Food insecurity was associated with lower CGPA and significantly higher odds of questioning both commitment to and ability to continue college education
**Umeda et al. (2024)**[90]		United States (public Hispanic-Serving Institution in Texas; Department of Kinesiology)	360 undergraduate students (final analytic sample)	Undergraduate university students (primarily upper-level kinesiology students)	USDA 10-item AFSSM [past 30 d; categorized as food secure (high/marginal) vs. food insecure (low/very low) following USDA scoring protocol]	• Self-reported CGPA	Food insecurity was significantly associated with lower CGPA, with the negative association more clearly observed among non-first-generation college students than first-generation students
**Weaver et al. (2020)**[24]		United States (Rowan University; midsized public research-intensive university in New Jersey)	2055 undergraduate students responded to the survey; GPA analyses conducted on a subsample of ∼1480 students with recorded GPAs	Undergraduate university students (second-year through senior students; first-year students excluded from GPA analyses)	USDA 10-item AFSSM (30-d reference period; 1 item omitted; summed score categorized as high, marginal, low, and very low food security; low + very low classified as food insecure)	• CGPA obtained from official university records (analyzed as lowest 10% vs. highest 10% GPA categories)	Food insecurity significantly increased the odds of being in the lowest 10% of GPA and significantly reduced the odds of being in the highest 10% of GPA, even after controlling for demographic and socioeconomic factors
**Wilcox et al. (2022)**[91]		United States (medium-size public state university in Louisiana, deep South region)	605 students (primary analytic sample); an additional subsample of 84 faculty, staff, and administrators included for perception analyses	Undergraduate and graduate university students (primary focus on students; employees analyzed separately)	Food insecurity questions adapted from the CUFBA toolkit, including self-reported experience of inadequate food supply during enrollment (yes/no) and frequency per academic term	• Self-reported CGPA	Food insecurity was significantly associated with lower self-reported GPA, as well as higher perceived stress and poorer physical and mental health outcomes
**Wood and Harris III (2022)**[92]		United States (7 community colleges in California)	4067 community college students	Community college students (undergraduate level)	Food insecurity measured as part of the CCSM, using a dichotomous indicator of food insecurity experienced within the past 2 y (food insecurity rarely occurred in isolation and was analyzed in combination with housing, transportation, and employment insecurities)	• Self-reported GPA	Students who did not experience food insecurity or other insecurities had significantly higher self-reported GPAs than students who experienced food insecurity in combination with other insecurities
**Zhong & Xie (2025)**[93]		United States (national samples of United States postsecondary institutions)	63,680 college students (spring 2020 n = 50,307; fall 2020 n = 13,373)	College students (undergraduate and graduate students enrolled in United States. 2-y and 4-y institutions)	USDA-based food insecurity items embedded in the ACHA–NCHA III survey (5-question version of the USDA Food Insecurity scale; 30-d reference period; categorized as high/marginal, low, and very low food security following ACHA scoring guidelines)	• Self-reported GPA	Food insecurity was significantly and negatively associated with self-reported GPA, even after controlling for mental health, stress, socioeconomic factors, and health conditions
**Longitudinal**							
**Cuite et al. (2023)**[44]	United States (public university in the Northeastern United States)		338 undergraduate students who completed surveys at both time points (2016 and 2019)	Undergraduate university students	USDA 10-item FSSM (2016) and USDA 18-item HFSSM (2019), 30-d reference period; categorized as high, marginal, low, and very low food security and dichotomized as food secure vs. food insecure	• CGPA from official university registrar records	Students who experienced food insecurity in 2016, 2019, or both time points had significantly lower CGPAs in 2019 than students who were food secure at both time points
**Collier et al. (2021)**[94]	United States (single 4-y public university in the Midwestern United States)		700 first-year students with complete survey data (693 included in GPA analyses)	Undergraduate university students (first-year students at a 4-y institution)	USDA 6-item short-form food security instrument (30-d reference period; categorized as high, marginal, low, and very low food security; marginal included in “any food insecurity”)	• CGPA from official university records (first-semester GPA) • Credits earned during the first semester (official university records)	Food insecurity was significantly associated with lower first-semester GPA and fewer credits earned, even after controlling demographics, prior achievement, and noncognitive factors
**Harville & King (2025)**[95]	United States (Midwestern colleges and universities in Illinois and Ohio)		Baseline: 551 college students; Follow-up: 131 students (longitudinal subsample)	Undergraduate college students	USDA 6-item short-form food security instrument (12-mo reference period; categorized as food secure vs. food insecure)	• Self-reported GPA	Food-insecure students had significantly lower mean self-reported GPAs than food-secure students at both baseline (2020–2021) and follow-up (2021–2022), although the longitudinal change in food security status itself was not statistically significant
**C. Leung et al. (2021)**[30]	United States. Nationally representative sample using the PSID		1574 individuals enrolled in college between 1999 and 2003 and followed through 2015–2019	College students aged 16–29 y during enrollment. Includes first-generation and economically independent students	18-item HFSSM measured during college enrollment (1999–2003). Classified as food secure vs. food insecure (score ≥1 at any wave)	• Educational attainment outcomes, including college completion and highest degree achieved (associate’s, bachelor’s, graduate/professional). Measured in adulthood (2015–2017)	Food insecurity during college was associated with significantly lower odds of college completion and lower likelihood of earning a bachelor’s or graduate degree
**Loofbourrow et al. (2023)**[96]	United States (University of California system; single large public university on a quarter system)		Cross-sectional quarterly samples ranged from 140 to 179 students; longitudinal subsample included 58 students.	Undergraduate and graduate university students	USDA 10-item AFSSM (30-d reference period; categorized as high, marginal, low, and very low food security; low and very low classified as food insecure)	• Quarter GPA from official university records	Greater food insecurity was significantly associated with lower quarter GPA, and participation in CalFresh (SNAP) significantly moderated this relationship by buffering the negative effect of food insecurity on GPA during the early COVID-19 pandemic
**Raskind et al. (2019)**[97]	United States (7 colleges and universities in Georgia)		2377 college and university students (analytic sample)	College and university students (undergraduate and technical college students aged 18–25 y)	USDA 6-item short-form FSSM(12-mo reference period; classified as food secure vs. food insecure following USDA scoring guidelines)	• Self-reported CGPA (treated as a continuous variable)	Food insecurity was significantly associated with lower GPA, and this association was fully mediated by poorer psychosocial health (depression, anxiety, and lower hope)
**Van Woerden et al. (2019)**[98]	United States (large public Research 1 university in the United States Southwest; Arizona State University)		591 first-year university students with complete food security, GPA, and background data	Undergraduate university students (first-year students at a 4-y institution)	USDA 6-item short-form food security instrument, adapted to a 1-mo reference period; students classified as food insecure if they reported ≥2 affirmative responses	• CGPA from official university records (fall 2015 and spring 2016 semesters) • University retention to fall 2016 (official enrollment records)	Food-insecure first-year students had significantly lower official GPAs than food-secure students even after controlling for high school academic performance and socioeconomic background, although food insecurity was not significantly associated with first-year retention
**Wolfson et al. (2022)**[20]	United States (nationally representative household panel survey)		1574 college students enrolled during 1999–2003 with longitudinal follow-up through 2015–2017	College students (undergraduate students enrolled in community colleges and 4-y institutions)	USDA 18-item HFSSM, measured during college enrollment (1999–2003); food insecurity defined as marginal, low, or very low food security at any point during college	• College graduation (degree completion) • Highest degree attained (no degree, associate’s, bachelor’s, graduate/professional), measured via follow-up surveys in 2015–2017	Experiencing food insecurity during college was significantly associated with lower odds of college graduation and a lower likelihood of obtaining a bachelor’s or graduate/professional degree, particularly among first-generation students

Abbreviations: ACHA, American College Health Association; AFSSM, Adult Food Security Survey Module; AFSS, Adult Food Security Screener; AG, academic grading; AP, Academic Performance; APS, Academic Progress Scale; BSW, Bachelor of Social Work; CCSM, Community College Success Measure; CGPA, cumulative grade point average; CPS-FSS, Current Population Survey Food Security Scale; CUFBA, College and University Food Bank Alliance; CUNY, City University of New York; ELCSA, Latin American and Caribbean Food Security Scale; FIES, Food Insecurity Experience Scale; FS, Food Security; FSSM, Food Security Survey Module; GPA, grade point average; HFIAS, Household Food Insecurity Access Scale; HFSSM, Household Food Security Scale Module; MSW, Master of Social Work; NCHA, National College Health Assessment; PSID, panel study of income dynamics; SNAP, Supplemental Nutrition Assistance Program; UC, University of California; UPM, Universiti Putra Malaysia, US-FSSM, United States Food Security Survey Module; WAM, weighted average marks.

**FIGURE 2:**
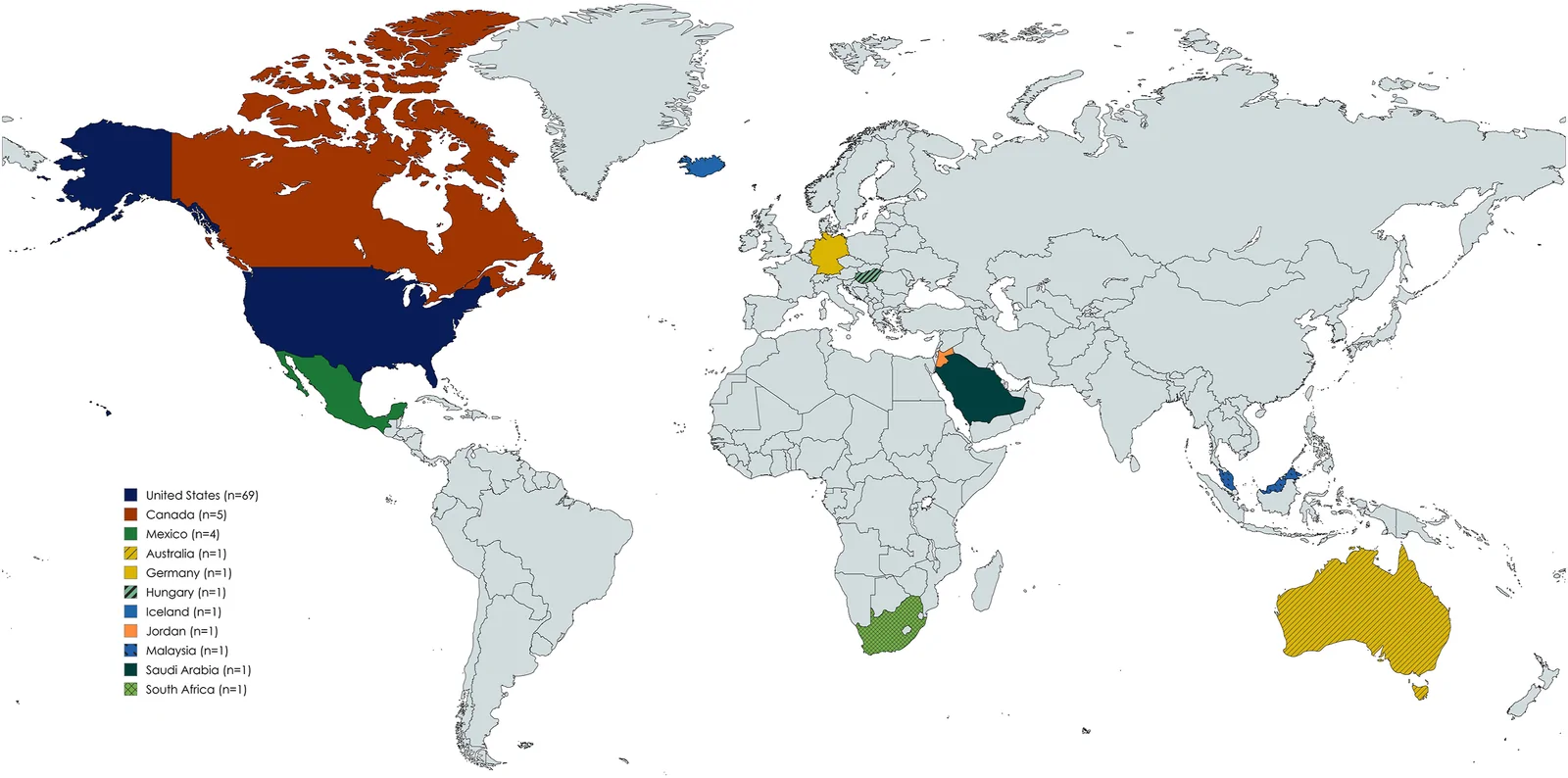
Geographic distribution of studies on food security and academic performance by country. Created with www.mapchart.net.

### Study settings

Among the 86 included studies, 69 (80.2%) were conducted in the United States and 17 (19.8%) were conducted in other countries. Among the United States studies, the predominant setting was public 4-y universities, including numerous large public research universities, state university systems, and land-grant institutions. Six studies specifically identified Hispanic-Serving and/or Minority-Serving Institutions, including institutions in California, New Mexico, and Texas. At least 5 studies explicitly identified land-grant universities, including institutions in Appalachia and the southeastern United States. Community colleges were represented in ≥9 studies, ranging from individual or multicampus community college systems to a study involving 113 community colleges across multiple states. At least 10 studies used multi-institutional, university system, or national samples, including University of California system-wide studies and samples spanning multiple colleges and universities across the United States. A smaller number examined specialized educational populations, including 1 nursing program, 1 dental school, 1 medical school, and 1 National Collegiate Athletic Association student-athlete setting. Geographically, studies encompassed urban, suburban, rural, Appalachian, border region, and regional institutions, although large public and urban universities were frequently represented.

### Student populations

The included studies examined a broad range of student populations enrolled in higher education institutions. Undergraduate students were the predominant population, appearing either exclusively or alongside graduate students in the large majority of studies. At least 26 United States studies included graduate or postgraduate students, although graduate students were often included within broader university samples rather than examined separately. Community college students were represented in ≥10 studies, including associate degree–seeking students, adult learners, and student veterans. Professional students were less frequently studied, with populations including nursing students, dental students, medical students, social work students, and law or other professional students. Several studies specifically targeted or described populations that may experience greater socioeconomic or structural vulnerability, including first-generation and nontraditional students, international students, undocumented students or students from mixed-status families, students of color, student-parents, Pell Grant recipients, commuter students, student-veterans, and students experiencing housing instability or compromised food access. However, these characteristics were not consistently reported or analyzed as distinct subgroups across studies. Overall, undergraduate students, particularly those attending United States public universities, were the most frequently studied population, whereas evidence specifically focused on graduate, professional, international, and other potentially vulnerable student populations was comparatively limited.

### Conceptual characteristics: measurement of food insecurity and academic outcomes

#### Measurement of food insecurity

Food insecurity was most commonly assessed using versions of the USDA Food Security Survey Modules, particularly the 10-item Adult Food Security Survey Module and the 6-item Short-Form Food Security Survey Module. Several studies also used the 18-item Household Food Security Survey Module (HFSSM), whereas others employed adapted or modified versions tailored for college student populations. Most studies categorized participants into levels of food security, including high, marginal, low, and very low food security, although many analyses further dichotomized participants as food secure compared with food insecure. The time frame used to assess food insecurity varied across studies. The most common reference period was the previous 12 mo, consistent with the standard USDA HFSSM format. However, several studies employed shorter recall periods, including past 30 d, past academic term, or current semester.

A smaller number of studies used alternative instruments, including the Food Insecurity Experience Scale, the Radimer–Cornell Food Insecurity Scale, the Latin American and Caribbean Food Security Scale, and measures informed by the Household Food Insecurity Access Scale. Several studies also used self-developed or study-specific food insecurity indicators assessing experiences such as worrying about food running out, skipping meals, or inability to afford balanced meals.

#### Measurement of academic performance

Academic performance was assessed using a wide range of self-reported and institutionally derived measures. The most commonly reported outcome was GPA or cumulative GPA (CGPA), which was frequently self-reported and occasionally obtained from official university or registrar records. Several studies categorized GPA or CGPA according to institutional grading thresholds or dichotomized students into higher compared with lower academic performance groups. In addition to GPA-based measures, many studies assessed broader indicators of academic functioning and disruption. These included self-reported concentration, attention in class, energy levels, comprehension of course material, class attendance, assignment completion, course withdrawal or failure, academic probation, delayed graduation, academic persistence, retention, and degree completion. Some studies also examined perceived impacts of food insecurity on academic success, including difficulties studying, reduced classroom engagement, and interruptions to educational progression.

Longitudinal studies more commonly incorporated objective academic outcomes obtained from institutional records, including semester GPA, credits earned, retention, graduation, and highest degree attained. Mixed-methods studies often combined GPA measures with self-reported perceptions of academic performance and educational challenges associated with food insecurity.

### Distribution of evidence by study design

#### Quantitative studies

##### Cross-sectional designs

A total of 59 cross-sectional studies examined the relationship between food insecurity and academic performance among postsecondary students (Table 2). These studies were conducted predominantly in the United States [24], [25], [28], [29], [39], [40], [41], [43], [49],[52], [53], [54], [55], [56], [57], [58], [59], [60], [63], [64], [65], [66], [67], [68], [69], [71], [72], [73], [74], [75], [76], [77], [78], [79], [80], [81], [82], [84], [85], [86], [87], [89], [90], [91], [92], [93], but also included studies from Malaysia [50], [51], [61], [88], Jordan [36], Saudi Arabia [37], Mexico [38], Canada [34], [62], Australia [35], South Africa [26], Iceland 83], and Germany [70]. Across these studies, academic performance was measured using a range of indicators, including GPA, CGPA, weighted average marks (WAM), self-reported grades, perceived academic decline, concentration, class attendance, course failure/withdrawal, academic progress, delayed graduation, and academic attrition. Overall, the cross-sectional evidence showed a consistent pattern of poorer academic outcomes among food-insecure students, although the strength of associations varied across studies and outcome measures.

#### GPA, grades, and academic achievement

A substantial proportion of cross-sectional studies assessed the relationship between food insecurity and GPA or grade-based academic outcomes, with the majority reporting a significant inverse association. Across diverse United States university settings, food-insecure students consistently demonstrated lower GPA and poor academic achievement than their food-secure peers. At the institutional level, multiple studies reported significant differences in GPA by food security status [24], [25], [28], [43], [54], [55], [56], [57], [59], [64], [67], [68], [71], [77], [81], [82], [87], [89], [91]. Food-insecure students generally had significantly lower GPA, ranging from 0.14 to 0.30 grade points lower than food-secure students [28], [43], [82], [91]. Food-insecure undergraduates experienced an increased likelihood of being in the bottom 10% of GPA rankings and a decreased likelihood of being in the top 10% [24]. Food-insecure students were noted to have nearly 6-fold higher odds of having a GPA of <2.5 [68]. In the California public university system, food insecurity was directly associated with lower GPA (*β* = −0.08, *P* < 0.001) and also indirectly associated with GPA through poor mental health [25].

Large-scale and multi-institutional studies also confirmed these findings [60], [65], [73], [74], [79], [92], [93]. In the National College Health Assessment III dataset spanning 75 United States universities, both low and very low food security were associated with reduced odds of achieving high GPA categories [73]. In this study, very low food security decreased the odds of attaining an A− or higher GPA by ∼41% compared with high or marginal food security [73]. Among first-year students across 8 United States universities, food insecurity was associated with significantly higher odds of having a GPA of <3.0 [odds ratio (OR) = 1.91, 95% CI: 1.19, 3.07] [60]. Similarly, in a large sample of >4000 students from 7 California colleges, students without any form of insecurity had significantly higher GPA than those experiencing food insecurity alongside other insecurities [92]. In community college settings, food insecurity was associated with lower GPA. However, this association was attenuated after adjustment for demographic factors such as race and living situation [74]. Further, data from 2 national surveys of United States college students showed that higher food insecurity scores were associated with lower GPA, and food insecurity remained a significant negative predictor of GPA after adjustment for mental health, stress, and other correlates (*β* = −0.10, *P* < 0.001) [93].

Studies that focused on specific student populations reported similar findings. Among Latinx immigration-impacted students in the University of California system, food insecurity was associated with a significant decline in GPA (*β* = −0.134) [66]. Among students utilizing campus food pantries across 9 University of California campuses, food insecurity was negatively associated with GPA (standardized *β* = −0.18) [41]. Further, food insecurity was also associated with lower GPA among nursing students [58], social work students [63], student veterans [40], and dental students reporting food/hunger-related stress [75].

Findings from other countries were generally similar. In Malaysia, food-insecure undergraduate students were less likely to achieve a high academic grade (≥3.7) than food-secure students [50], and food insecurity was negatively correlated with academic performance in mediation analyses [51]. Other Malaysian studies also reported that food insecurity was significantly associated with CGPA [88], [61]. In Atlantic Canada, food-insecure students were more likely to report poorer GPAs, and poorer academic performance significantly predicted greater psychological distress, particularly among food-insecure students [34]. In Iceland, food-secure students were less likely to report negative academic performance and nearly twice as likely to report positive or neutral academic outcomes compared with food-insecure students [83]. Similarly, among postgraduate students in Jordan, average GPA was highest among students with high food security (∼91%), lower among those with marginal food security (∼86%), and lowest among those with low or very low food security (∼79%) [36]. In Saudi Arabia, the correlation between food security and academic performance was positive but not statistically significant [37].

#### Course failure, withdrawal, attrition, and delayed graduation

Beyond GPA, several American studies showed that food insecurity was associated with course-level and enrollment-related academic difficulties [28], [29], [68], [72], [78], [80], [82], [85], [86]. Food-insecure students have been noted to be twice as likely to fail a course, almost twice as likely to withdraw, 4 times more likely to take an incomplete compared with food-secure students [28], and more likely to suspend their studies or forgo purchasing required course materials due to finances [68]. Students experiencing food insecurity also had higher odds of dropping out, withdrawing from classes [75], [82], considering dropping out, reducing their course load, and neglecting their academic studies because of debt-related financial strain [82]. The combination of food insecurity and housing instability more than doubled the likelihood of dropping out compared with students who had secure access to both food and housing [85]. Further, a recent study at a large research-intensive Hispanic-Serving Institution in the United States–Mexico border region found that low food security was associated with increased delays in student graduation [80]. Taken together, these findings indicate that food insecurity does not just affect grades; it also increases the chances of course failure, withdrawal, attrition, and delayed graduation, which highlights how unmet basic needs can disrupt students’ ability to complete their degrees.

#### Perceived academic performance, concentration, and academic progress

Several studies found that food insecurity was associated with poorer concentration, lower energy, and reduced perceived academic progress [26], [38], [54], [64], [65], [75], [76], [77], [80], [87], [62]. In a cross-sectional survey of students across 10 public universities in the Appalachian and Southeastern United States, food insecurity was found to be significantly associated with lower academic progress scores [65]. Another study focusing on dental students found that 33% reported experiencing study or academic performance difficulties linked to food or hunger concerns [75]. In Appalachia, food-insecure students were less likely to rate their school progress, attendance, attention span, and understanding of concepts favorably [77]. A United States study of community college students found significant relationships between food insecurity and GPA, concentration, and energy [87]. A large California-based survey found that students with food insecurity were more likely to report lower academic performance and difficulty concentrating [76]. Conversely, higher food security has been strongly associated with better well-being outcomes, including higher energy levels, better overall health, and improved concentration among both undergraduate and graduate students [54]. These findings have also been confirmed in other countries. In Canada, severe food insecurity was associated with an inability to concentrate on class or during examinations [62]. In Mexico, a tendency toward higher grades among food-secure students was noted, and food insecurity was significantly associated with poorer perceptions of academic progress, attendance at classes, ability to pay attention in class, understanding concepts taught, and academic future [38]. Among South African students, hunger reduced concentration and vigor and contributed to missed classes [26]. Overall, the evidence consistently suggests that food insecurity is linked to weaker perceived academic performance and diminished day-to-day cognitive and physical functioning needed for learning.

#### COVID-19 pandemic context

Several cross-sectional studies highlighted the impact of the COVID-19 pandemic on the relationship between food insecurity and academic outcomes among university students [29], [39], [49], [52], [53], [69], [70], [83], [88], [90], [93]. In the United States, food-insecure students consistently reported greater perceived declines in academic performance than their food-secure peers during the COVID-19 pandemic. For instance, among married student households, a significantly higher proportion of food-insecure households with children reported worsened academic performance during the pandemic (60.0% compared with 28.0%, *P* = 0.027) [49]. Evidence from a Hispanic-serving and minority-serving institution further showed that food-insecure students were more likely to report worsened grades during the pandemic compared with food-secure students (60.5% compared with 38.6%, *P* < 0.05), particularly among racial/ethnic minority female students [53]. Food insecurity was associated with reduced ability to perform schoolwork, increased likelihood of course withdrawal, and anticipated delays in graduation during COVID-19 disruptions [29]. In addition, worsening food security status during the pandemic was significantly associated with lower GPAs [39]. Similar patterns were observed outside the United States. In Germany, food-insecure students were significantly more likely to report negative impacts of the pandemic on academic or research progression compared with food-secure students (70.7% compared with 51.6%, *P* < 0.0001) [70]. Similarly, both academic performance and CGPA were significantly related to food insecurity among Malaysian students [88]. Collectively, these studies suggest that the pandemic context intensified the adverse relationship between food insecurity and academic performance, particularly through disruptions to learning, increased financial strain, and broader psychosocial challenges.

#### Studies reporting null or mixed findings

Although the overall trend pointed toward poor academic outcomes among food-insecure students, a few studies reported null or mixed findings, particularly when academic performance was assessed only by GPA. For example, no significant association between food insecurity and GPA was found in some United States studies [53], [81], [84] and in 1 Mexican study, despite significant associations with other academic-function indicators [38]. In a community college sample, food insecurity was not significantly associated with GPA across all GPA categories, although significant differences emerged between selected GPA categories, and the association attenuated after adjustment for race and living situation [74]. In Saudi Arabia, the relationship between food security and academic performance was positive but not statistically significant [37]. In another study, a negative correlation between food insecurity and GPA was statistically significant in 1 semester but only trended in the same direction in the other 2 semesters [56]. Some studies also found significant associations for perceived worsening of grades or broader academic difficulties even when GPA itself was not statistically different [53], [81].

#### Mediation by psychological distress and related mental health factors

Several cross-sectional studies examined whether mental health mediated the relationship between food insecurity and academic performance [29], [35], [67], [81], [93], [51]. Among Malaysian students, anxiety and depression both mediated the relationship between food insecurity and academic performance [51]. Similarly, in the United States, psychological well-being and sleep were reported to mediate the relationship between food security and GPA [67]. However, a different United States study found that food-insecure students reported more depressive symptoms and greater academic disruption, although GPA did not differ by food security status, which suggests an indirect pathway through psychological well-being rather than a direct effect on performance [81]. During the pandemic, anxiety and depression partially mediated the relationship between food insecurity and decreased ability to do schoolwork, whereas depression mediated delayed or uncertain graduation [29]. In Australia, psychological distress partially mediated the negative relationship between food insecurity and WAM [35]. Nationally representative American data indicate that food insecurity remains a significant negative predictor of GPA even after controlling for psychological well-being and related mental health factors [93]. Furthermore, across studies, food insecurity was consistently related to poorer psychosocial health, including higher stress, anxiety, depression, and distress [34], [38], [50], [56], [60], [66], [68], [71], [91]. Overall, the evidence suggests that mental health and psychological well-being may act as either full or partial mediators in the relationship between food insecurity and academic performance.

#### Longitudinal designs

A smaller but important body of research examined the relationship between food insecurity and academic outcomes over time [20], [30], [44], [94], [95], [96], [97], [98] (Table 2). All identified longitudinal studies were conducted in the United States. Several longitudinal studies demonstrated that food insecurity predicts poor academic performance. In a prospective cohort study of first-year and transfer students at a 4-year university, food insecurity measured at the beginning of the semester was significantly associated with lower first-semester GPA and fewer credits earned after adjustment for demographic, academic, and psychosocial characteristics [94]. Similarly, among first-year university students, food-insecure students had significantly lower GPA across both the Fall 2015 and Spring 2016 semesters, even after accounting for high school academic performance and socioeconomic background [98]. The authors also estimated that the proportion of food-insecure students achieving at least a “B” average would increase from 59% to 72% if these students were food secure. Although food-insecure students were less likely to remain enrolled in the subsequent academic year, this difference did not reach statistical significance [98]. Longitudinal evidence also suggests that food insecurity may persist or worsen over time and continue to negatively influence academic achievement. In a repeated-survey study conducted between 2016 and 2019 at a public university, the prevalence of food insecurity increased from 22.4% to 41.0%, with nearly half of students (47%) experiencing food insecurity at least once during the study period [44]. Students experiencing food insecurity at either time point had significantly lower GPA at the second measurement point, whereas first-generation students were consistently at greater risk of worsening food security status over time [44]. During the COVID-19 pandemic, food insecurity among college students increased from 31.3% to 39.7% over 2 y. In the same study, food-insecure students had lower mean GPA and higher levels of depression and perceived stress than food-secure students across follow-up periods [95].

Beyond short-term academic performance, nationally representative longitudinal studies demonstrated that food insecurity during college may have enduring educational and socioeconomic consequences. Using the Panel Study of Income Dynamics data, following college students from 1999 to 2003 through 2015 to 2017, Wolfson et al. (2022) found that food insecurity during college was associated with significantly lower odds of college graduation (OR = 0.57, 95% CI: 0.37, 0.88), especially among first-generation students [20]. Food insecurity during college has not only been associated with lower educational attainment and college completion but also with lower subsequent labor income, reduced wealth accumulation, and greater likelihood of experiencing food insecurity later in adulthood [30]. Together, these findings suggest that food insecurity during the college years may contribute to long-term educational and economic disadvantage.

Multiple longitudinal studies identified psychosocial factors as important mechanisms linking food insecurity to academic performance. In a 2-y cohort study, food insecurity was associated with poor psychosocial health, including depression, anxiety, and reduced hopefulness, which in turn predicted lower GPA [97]. Structural equation modeling indicated that psychosocial health mediated ∼73% of the association between food insecurity and GPA, and the direct effect of food insecurity on GPA was no longer significant after accounting for psychosocial factors [97]. Likewise, Collier et al. [94] found that food insecurity was associated with increased financial and psychological distress and greater academic amotivation, alongside lower GPA and credits earned.

One study also examined the potential buffering effects of food assistance programs [96]. This longitudinal analysis conducted during the 2020–2021 academic year found that participation in the Supplemental Nutrition Assistance Program (SNAP; known as CalFresh in California) moderated the relationship between food insecurity and GPA [96]. Specifically, the interaction between food insecurity and CalFresh participation was positively associated with GPA, which suggests that food assistance participation may help mitigate some of the adverse academic consequences of food insecurity. Overall, the longitudinal evidence supports the findings from cross-sectional studies and strengthens the temporal relationship between food insecurity and poor academic outcomes among university students.

#### Qualitative studies

Eleven qualitative studies were identified that explored students’ lived experiences of food insecurity and its perceived influence on academic functioning and broader educational experiences (Table 3). Eight studies were conducted in the United States [32], [33], [99], [100], [101], [102], [104], [105], 1 in Canada [103], and 1 in Hungary [42]. The qualitative studies included diverse postsecondary student populations, such as college athletes [99], nontraditional students [32], general undergraduate student populations [33], [105], [104], medical students [102], and international students [42], [103]. Most studies employed individual interviews as the primary method of data collection to explore participants’ experiences in depth. One study adopted a modified PhotoVoice methodology, whereby participants documented their experiences through photographs and subsequently engaged in one-on-one interviews to contextualize and reflect on the images [101]. Across studies, students consistently described food insecurity as affecting academic performance through multiple interconnected pathways. Six overarching thematic domains were identified: *1*) impaired concentration and cognitive function; *2*) physical fatigue and low energy; *3*) psychological distress and mental health strain; *4*) reduced academic engagement and attendance; *5*) time scarcity and work–study trade-offs; and *6*) coping strategies with academic consequences.

**TABLE 3:** Characteristics of the included qualitative studies

Author (y)	Country setting	Sample size (n)	Population studied	Qualitative design	Analytic approach	Main qualitative themes/findings
Allen & Alleman (2019) [31]	United States; private, selective, affluent research university	10 food-insecure students	Undergraduate students experiencing severe food insecurity	Interpretative phenomenological qualitative interview study	2-cycle analysis (in vivo coding followed by interpretive phenomenological analysis); NVivo; member checking and intercoder reliability procedures	• Hunger and inadequate nutrition impairing concentration and learning • Health-related symptoms such as fatigue, migraines, and fainting affecting academic participation • Stress and worry about food reducing time and mental capacity for studying • Academic decisions shaped by food and financial constraints • Interruptions to academic progression, including reduced course loads, taking semesters off, transferring institutions, or leaving extracurricular activities
Anziano & Zigmont (2024) [99]	United States; public university in New England (NCAA athletics)	10 food-insecure NCAA student-athletes	Undergraduate NCAA student-athletes	Generic qualitative interview study	Thematic analysis with constant comparison; dual coding and consensus; themes organized using a socioecological framework	• Difficulty concentrating on class • Reduced energy affecting academic engagement
Beam (2020) [32]	United States; public 4-y university (Midwest)	8 food-insecure undergraduate students	Nontraditional undergraduate students (e.g., working students, student-parents, financially independent)	Qualitative case study using semistructured interviews	Thematic content analysis; iterative coding with member checking	• Missed classes due to work or transportation constraints • Financial trade-offs between food, transportation, and attending class • Reduced concentration in lectures and examinations • Impaired learning and missed academic content • Difficulty affording textbooks and academic materials • Academic delays including incompletes due to cost barriers
Fernandez et al. (2019) [100]	United States; community colleges and 4-y institutions (Texas and Florida)	36 food-insecure undergraduate students (longitudinal interviews)	Undergraduate college students (high proportion first-generation and nontraditional)	Longitudinal qualitative interview study	Hybrid deductive–inductive thematic analysis using constant comparison across interview waves	• Chronic stress from balancing work, academics, and expenses • Sleep disruption and poor diet contributing to illness
Gahan et al. (2022) [101]	United States; large public research university (Midwest)	10 food-insecure undergraduate students	Undergraduate university students (diverse racial/ethnic backgrounds)	Qualitative interview study with photo elicitation	Inductive thematic analysis with team-based coding and consensus	• Distraction from hunger during studying and examinations • Impaired concentration in class • Reduced ability to focus due to physiological hunger and food-related stress
Gamba et al. (2021) [33]	United States; large public urban university (West Coast)	30 college students with marginal food security	Primarily undergraduate students (racially/ethnically diverse)	Qualitative descriptive interview study	Inductive thematic analysis using team-based coding and consensus	• Competing demands between school, work, and eating • Fatigue, headaches, and low energy from inadequate or unbalanced diets • Impaired concentration and class participation due to poor nutrition
Hammad et al. (2025) [102]	United States; private medical school, Boston, MA	25 food-insecure medical students	Graduate medical students	Qualitative descriptive interview study	Inductive thematic analysis with team-based coding and consensus (COREQ-guided)	• Increased fatigue and reduced energy • Difficulty concentrating during classes and hospital rotations • Performance impacts linked to skipping meals or inadequate nutrition • Illness-related absenteeism
Hanbazaza et al. (2021) [103]	Canada; large public university (Western Canada)	11 food-insecure international students	International postsecondary students (primarily graduate)	Qualitative descriptive interview study	Inductive manifest content analysis with iterative coding	• Lack of concentration • Missing classes and examinations
Hilal et al. (2025) [42]	Hungary; large public research university	15 food-insecure international students	International undergraduate and postgraduate students	Qualitative descriptive interview study	General inductive analysis with dual coding and consensus	• Difficulty concentrating • Sacrificing studies for work • Diminished academic performance • Loss of motivation and interest in studies
Meza et al. (2019) [104]	United States; large public research university (California)	25 food-insecure undergraduate students	Undergraduate students (predominantly first-generation and students of color)	Qualitative descriptive interview study	General inductive thematic analysis with team-based coding and consensus	• Physical manifestations of food insecurity impact academic performance • Mental trade-off between thinking about food and studying
Zigmont et al. (2021) [105]	United States; mid-sized public university (New England)	19 food-insecure undergraduate students	Undergraduate university students (predominantly commuters)	Generic qualitative study using surveys and interviews	Thematic analysis with constant comparison and dual coding	• High levels of stress and worry about accessing food • Distraction from academic tasks due to food-related concerns • Difficulty concentrating • Physical symptoms such as tiredness, hunger, and weight loss • Emotional strain affecting academic engagement

Abbreviations: COREQ, Consolidated Criteria for Reporting Qualitative Research; NCAA, National Collegiate Athletic Association.

### Theme 1: impaired concentration and cognitive function

The most consistently reported academic consequence of food insecurity was difficulty concentrating on academic tasks. Across studies, students described hunger and preoccupation with food as barriers to paying attention in class, studying effectively, and completing examinations [31], [32], [33], [42], [99], [101], [102], [103], [104], [105]. In a PhotoVoice study, students reported that hunger distracted them while studying and during examinations [101]. Similarly, students on the United States West Coast reported that skipping meals or eating poorly reduced their ability to pay attention and participate in class [33]. Among food-insecure students recruited from a campus food pantry in California, participants described a “mental trade-off” between focusing on food and focusing on academics, whereby concerns about where and how to obtain food competed directly with attention to coursework [104].

This theme was also evident among international students. In Canada, international students using an on-campus food bank reported that lack of food impaired concentration during lectures, studying, and examinations; several students linked hunger to impatience, distraction, and difficulty remaining engaged in class [103]. Similarly, international students in Hungary described difficulty concentrating as one of the primary academic implications of food insecurity [42]. Among collegiate athletes, food insecurity was reported to affect classroom concentration, with participants describing low energy and reduced ability to focus in class when they had not eaten enough [99]. Collectively, these findings suggest that impaired concentration is a central pathway through which food insecurity affects academic functioning.

### Theme 2: physical fatigue and low energy

A second recurring theme was the role of fatigue, low energy, and physical symptoms in limiting academic performance. Students commonly described hunger, skipped meals, and poor diet quality as producing tiredness, headaches, weakness, sluggishness, and reduced stamina [31], [33], [105], [102], [99]. In a study by Meza et al. [104], students reported that inadequate food intake reduced their energy to study and perform well in school; for some, the loss of stamina was severe enough to prompt thoughts of changing majors or leaving demanding programs. Gamba et al. [33] similarly found that students attributed headaches, grogginess, bloating, and low energy to missed meals or nutritionally inadequate diets, and these physical symptoms interfered with schoolwork and class participation.

Among medical students, food insecurity contributed to fatigue and difficulty functioning during classes and clinical rotations. Participants described the physical demands of rotations as especially challenging when they were skipping meals or prioritizing food cost over nutritional adequacy [102]. International students in Canada also reported that hunger and inadequate food reduced their energy to study and contributed to missed classes and examinations [103]. These findings indicate that the physical manifestations of food insecurity may compromise students’ capacity to sustain attention, attend class, complete academic work, and meet the demands of intensive academic programs.

### Theme 3: psychological distress and mental health strain

Qualitative studies also emphasized that food insecurity created persistent psychological distress, including worry, stress, anxiety, embarrassment, and frustration [31], [32], [105], [100]. Students described constantly thinking about budgets, food availability, bills, and basic needs, which interfered with learning and academic performance. In a study by Beam [32], students explained that financial stress and worries about groceries, bills, and future food access were distracting during lectures and examinations. Several students reported mentally reworking budgets or checking bank accounts during class, resulting in missed course material and poorer performance on assignments or examinations. Fernandez et al. [100] similarly described food-insecure students as experiencing unrelenting stress related to employment, academic expectations, and uncertain control over expenses. This stress was often compounded by poor diet, lack of sleep, illness, and family obligations, creating a cycle that disrupted academic progress. Zigmont et al. [105] also found that students viewed food insecurity as a major source of stress and worry, with hunger and concerns about obtaining food making it difficult to focus on school. These studies suggest that the psychological burden of food insecurity may be as academically disruptive as hunger itself, particularly when stress becomes chronic and competes with students’ cognitive and emotional resources.

### Theme 4: reduced academic engagement, attendance, and performance

Several qualitative studies showed that food insecurity affected not only students’ capacity to concentrate but also their academic engagement, attendance, and perceived performance [31], [33], [42], [102], [103]. Students reported missing classes, withdrawing from courses, falling behind, reducing course loads, or avoiding academic activities because of food-related financial pressures. In a study by Beam [32], students described missing class to work additional hours, staying home to save gas money for food, or bringing children to class because they could not afford transportation and childcare. Others reported difficulty purchasing textbooks or academic materials. Among international students in Canada, food insecurity was linked to missed classes, missed examinations, and course withdrawal [103]. Students described being too hungry or low in energy to study, attend class, or complete assessments successfully [103]. In a study by Hilal et al. [42], students identified diminished academic performance and loss of motivation and interest in studies as key academic consequences of food insecurity. In a study by Hammad et al. [102], medical students described food insecurity as affecting performance in clinical rotations and, in some cases, causing students to fall behind when illness or fatigue prevented attendance. These findings demonstrate that food insecurity can disrupt academic engagement at multiple levels, from momentary in-class attention to course completion and persistence.

### Theme 5: time scarcity and work–study trade-offs

Another common theme was the trade-off between meeting basic needs and fulfilling academic responsibilities. Students frequently described working long hours to afford food, housing, tuition, and other expenses, which resulted in less time and energy for studying, attending class, or participating in campus life. In a study by Beam [32], students reported missing class to pick up work hours or making decisions between transportation to class and having money for dinner. Fernandez et al. [100] similarly noted that students with low food security faced competing demands from work, academics, expenses, and family support networks, all of which could disrupt academic progress. The time burden of meeting basic needs was also evident in a study by Meza et al. [104], where students reported working additional hours to cover living expenses after paying for housing, utilities, and tuition, resulting in fewer hours available for studying. In a study by Hilal et al. [42], sacrificing studies for work emerged as a key academic implication of food insecurity among international students. These studies highlight how food insecurity is often embedded within broader financial precarity, forcing students to make trade-offs that reduce time for learning and academic engagement.

### Theme 6: coping strategies with academic consequences

Finally, qualitative studies described a range of coping strategies that helped students manage food shortages but often carried academic costs [32], [33], [104], [102]. Students reported skipping meals, eating inexpensive and less nutritious foods, relying on campus food pantries, using social networks, sleeping to cope with hunger, or prioritizing educational expenses over food. Although these strategies sometimes helped students remain enrolled, they also contributed to fatigue, poor diet quality, stress, stigma, and reduced academic capacity. In a study by Gamba et al. [33], students described purchasing inexpensive foods, fast food, and shelf-stable staples because healthier options were unaffordable. These dietary compromises contributed to physical discomfort, poor sleep, and reduced ability to study. Meza et al. [104] found that some students slept for long periods as a response to hunger or experienced sleep deprivation because they were preoccupied with food. Beam [32] documented trade-offs between food and academic expenses, including textbooks, transportation, and professional school application fees. Students described choosing between paying for food and investing in academic success. These findings suggest that coping strategies may mitigate immediate hunger but can also produce longer-term academic consequences by reinforcing stress, reducing diet quality, limiting study time, and delaying the use of available supports.

Overall, qualitative studies provided detailed insight into the mechanisms through which food insecurity affects academic performance among postsecondary students. Students consistently described food insecurity as interfering with academic success through hunger-related distraction, fatigue, psychological distress, missed classes, reduced participation, work–study disagreement, and difficult trade-offs between food and educational expenses.

#### Mixed-methods studies

A total of 8 mixed-methods studies examined the relationship between food insecurity and academic performance among postsecondary students (Table 4) [45], [106], [107], [108], [109], [110], [111], [112]. These studies were conducted in the United States (*n* = 6) and Canada (*n* = 2) and combined quantitative survey findings with qualitative narratives or interviews. Across studies, the qualitative findings consistently complemented the quantitative results by illustrating the day-to-day academic consequences of food insecurity, including difficulty concentrating, fatigue, psychological stress, time scarcity, reduced engagement, and trade-offs between employment and academic responsibilities.

**TABLE 4:** Characteristics of the included mixed-methods design studies

Author (y)	Country/setting	Sample size	Population studied	FS measure	AP measure	Key findings
**Quantitative component**						
**Frank (2018)**[45]	Canada. Primarily undergraduate rural university in Nova Scotia	1030	Undergraduate and graduate university students	HFSSM. 10-item adult scale adapted for students. Classified as food secure, moderately food insecure, severely food insecure	Self-reported overall grades. Dichotomized as primarily A grades vs. primarily C, D, or F grades	Severely food-insecure students were >5 times more likely to report poorer grades than food-secure students after adjustment for sociodemographic factors
**Hickey et al. (2019)**[106]	United States. Liberal art college in New Hampshire	371	Undergraduate college students across disciplines. Majority student-athletes	Perceived hunger measured using a study-specific survey informed by food insecurity literature.	Self-reported GPA. Self-reported academic impact of hunger using a Likert-scale item. “Has hunger caused you not to perform as well in a class as you otherwise could have?”	36.4% of students reported that hunger negatively affected academic performance. Students who self-reported hunger had significantly lower GPAs than those who did not report hunger (*P* = 0.008)
**Hall et al. (2024)**[107]	United States (large southeastern land-grant university; Virginia Tech)	2116 (6% response rate from 35,337 invited)	Undergraduate and graduate students aged ≥18 y enrolled at the university	Modified USDA 10-item HFSSM (12-mo recall; adapted wording for college students; categorized as high/marginal vs. h low/very low food security)	Self-reported CGPA (categorized; < 3.0 vs. ≥3.0 used in analyses)	Students with GPAs below 3.0 were significantly more likely to experience low or very low food security, with food insecurity clustering alongside poor mental health, housing instability, and financial strain
**Khosla et al. (2020)**[108]	United States. Large, diverse public university. California State University, East Bay	53 at baseline. 48 completed all waves	Undergraduate students purposively recruited for likelihood of food insecurity, housing instability, or related challenges	Self-reported food insecurity indicators using 30-d items. Worried food would run out. Food purchased did not last. Rated impact of food access on academic progress on a 0–5 scale	Academic goal attainment using Goal Attainment Scaling. Self-defined academic goals.	Students experiencing food insecurity reported greater challenges to achieving academic goals
**Maynard et al. (2018)**[109]	Canada. University of Waterloo	14	Undergraduate university students living off campus and experiencing compromised food access	HFSSM, adult 10-item scale, classified as marginal, moderate, or severe food insecurity	Academic performance assessed via self-reported perceived academic impact on survey and interviews	Students experiencing marginal to severe food insecurity reported perceived negative effects on academic success
**Spaid & Gillett-Karam (2018)**[110]	United States. 4 community colleges in a Mid-Atlantic state	217	Community college students. Majority females, including single parents and Pell Grant recipients	Modified HFSSM. 8 items used to classify students as food secure vs. food insecure	Self-reported GPA, energy level, and concentration level as indicators of student success	Food insecurity was linked to indicators associated with poorer academic performance and completion potential
**Ahmed et al. (2025)**[111]	United States. Public 4-y college system in New York City (CUNY)	246	Undergraduate college students during the first year of the COVID-19 pandemic	USDA 10-item AFSSM. Categorized as high, marginal, low, and very low food security	Self-reported GPA. Composite scale assessing impact of food insecurity on academics including difficulty concentrating, studying, completing assignments, attending class, and course withdrawal	Students with low or very low food security had significantly lower GPA than food-secure students. Food insecurity was associated with greater interference with concentration, studying, class attendance, and academic performance overall
**Weaver et al. (2022)**[112]	United States. Public comprehensive university in the Northeast	1071 students with classifiable food security status	Undergraduate university students	USDA 10-item AFSSM, adapted to a 30-d reference period. Categorized as high, marginal, low, or very low food security	Institutionally linked GPA	Students with very low food security had significantly lower GPA than students with high food security
**Author** (y)	Country/setting	Sample size (n)	Population studied	Qualitative design	Analytic approach	Main qualitative themes/findings
**Qualitative** component						
**Frank (2018)**[45]	Canada; rural public undergraduate university (Nova Scotia)	1030	Undergraduate university students experiencing food insecurity	Embedded qualitative analysis of open-ended survey responses	Inductive thematic summarization; qualitative procedures not explicitly described	• Financial strain and unmet basic needs • Psychological stress and health burden • Time loss from learning due to work and food acquisition
**Hickey et al. (2019)**[106]	United States; small public liberal art college (New England)	210	Undergraduate college students (including student-athletes)	Embedded qualitative analysis of open-ended survey responses	Thematic summarization of written responses; analytic procedures not explicitly described	• Stigma and shame related to pantry use • Perceived undeservingness and reluctance to seek help
**Hall et al. (2024)**[107]	United States; large southeastern land-grant university (Virginia Tech)	176 written qualitative responses (within a survey of 2116 students)	Undergraduate and graduate students aged ≥18 y enrolled at the university	Mixed-methods study with an embedded qualitative component using open-ended survey responses	Inductive thematic coding conducted independently by 3 researchers and refined through group discussion	• Increased stress, fatigue, and difficulty concentrating • Reduced time and energy for coursework • Food-related stress hindered academic engagement
**Khosla et al. (2020)**[108]	United States; large public comprehensive university (California)	48	Undergraduate students experiencing food and housing insecurity	Longitudinal qualitative interview study embedded in mixed-methods design	Iterative descriptive and pattern coding using Dedoose	• Basic needs insecurity as a barrier to academic goals • Time scarcity and work–study conflict • Cognitive and emotional burden
**Maynard et al. (2018)**[109]	Canada; large public research university (Ontario)	14 food-insecure students	Undergraduate students (off-campus; domestic and international)	Qualitative interview study embedded in mixed-methods design	Hybrid deductive–inductive thematic analysis guided by adaptive theory and constant comparison	• Difficulty concentrating on class and while studying • Hunger and low energy • Reduced ability to complete assignments and perform well on examinations • Relying on low-cost foods • Food-related stress and anxiety interfered with learning
**Spaid & Gillett-Karam (2018)**[110]	United States; community colleges (mid-Atlantic region)	22 administrators (16 phase 1; 6 phase 2 interviews)	Community college administrators and food pantry leaders	Multistage qualitative interview study embedded in mixed-methods design	Aggregated thematic analysis with inductive coding to identify common patterns across interviews	• Hunger was perceived to place students at academic risk
**Ahmed et al. (2025)**[111]	United States; large public urban university (CUNY system)	246 (written narratives)	Undergraduate students (predominantly students of color and lower-income)	Narrative qualitative analysis embedded in mixed-methods design	Hybrid inductive–deductive narrative analysis with team coding and consensus	• Financial and structural barriers to accessing food contributed to challenges in achieving academic goals
**Weaver et al. (2022)**[112]	United States; mid-sized public university (Northeast)	1374 qualitative comments	Undergraduate university students	Embedded qualitative analysis of open-ended survey responses	Phenomenological thematic analysis using NVivo with team coding and constant comparison	• Time and convenience constraints • Difficulty balancing food needs with educational expenses • Delayed or forgone textbook purchases • Reduced concentration and ability to focus on studies • Skipping meals and reducing food intake

Abbreviations: AFSSM, Adult Food Security Survey Module; AP, Academic performance; CGPA, cumulative grade point average; CUNY, City University of New York; FS, Food security; GPA, grade point average; HFSSM, Household Food Security Scale Module.

Several mixed-methods studies identified significant quantitative associations between food insecurity and poor academic performance. In rural Canada, food-insecure students reported significantly worse academic performance, alongside poor mental health and higher stress [45]. Similarly, in the southeastern United States, students with GPA <3.0 were ∼2.6 times more likely to experience low food security compared with students with GPA between 3.5 and 4.0 [107]. In other United States studies, students reporting hunger had significantly lower self-reported GPA than those not reporting hunger [106] and reported greater academic difficulties [111]. Several studies also emphasized the effects of food insecurity on broader indicators of student success beyond GPA. Among community college students, food insecurity was significantly associated with lower energy levels, poorer concentration, and challenges related to student success [110]. Similarly, Weaver et al. [112] found that students with very low food security frequently struggled to balance educational expenses with food needs, often sacrificing textbooks, school supplies, or dietary quality to remain enrolled. In a longitudinal mixed-methods study, students described how food and housing insecurity, work–school disagreement, and financial strain interfered with their academic progress and ability to achieve educational goals [108].

In alignment with the qualitative studies, the qualitative findings across mixed-methods studies revealed several recurring mechanisms through which food insecurity affects academic functioning. Students commonly described difficulties concentrating on class, studying, and completing assignments when experiencing hunger or food shortages. Canadian students explained that inadequate food access hindered their ability to focus academically and negatively affected examination preparation, studying, and classroom attention [109]. Similarly, students from rural Nova Scotia described spending excessive time attempting to meet basic needs, particularly through paid employment, at the expense of time available for academic engagement [45]. United States students also reported working long hours to meet food and housing costs, with some indicating that employment demands substantially reduced their ability to achieve stronger academic performance [108].

Food insecurity was also closely tied to psychological distress and emotional burden within qualitative narratives. Students described persistent stress, anxiety, and worry related to obtaining food, often alongside existing academic pressures. In a study by Maynard et al. [109], participants characterized the experience as “stress plus stress,” which referred to the combined burden of academic responsibilities and food-related concerns. Students in a study by Hall et al. [107] similarly described exhaustion, stress, and deteriorating grades associated with balancing work obligations, financial hardship, and food insecurity. Ahmed et al. [111] further reported that food-insecure students frequently reflected on financial hardship, inadequate institutional support, and food program reliance in their narratives.

Another recurring qualitative theme from mixed-methods studies involved coping strategies and compromises that carried academic consequences. Students reported relying on inexpensive, low-quality foods, skipping meals, delaying textbook purchases, reducing course loads, or increasing work hours to afford food and housing [45], [108], [109], [112]. In some cases, students reported interrupting their studies or transitioning to part-time enrollment because of financial strain and food insecurity [109]. Students also described stigma related to food pantry use and reluctance to access available supports, despite experiencing hunger that affected academic functioning [106], [112]. Overall, the mixed-methods literature highlighted that the impact of food insecurity on academic performance extends beyond GPA and shapes students’ broader capacity to engage, persist, and succeed in higher education.

### Evidence gaps and research opportunities

Despite the growing body of literature examining the relationship between food insecurity and academic performance among postsecondary students, several important evidence gaps remain.

### Geographic and population gaps

The existing evidence is heavily concentrated in the United States, which accounted for the vast majority of included studies. Of the 86 studies, 69 were conducted in the United States, whereas relatively few studies originated from Canada (*n* = 5), Malaysia (*n* = 4), and other countries. This geographic concentration limits the generalizability of findings to higher education systems operating in different economic, cultural, and policy contexts. Additional research is needed in low- and middle-income countries and in understudied regions, including Latin America, Africa, Asia, and Europe. Further, among the 69 studies conducted in the United States, the literature represented all major United States regions, including the Northeast, Midwest, South and Southeast, and West, as well as national and multistate samples. However, the distribution of studies was uneven, with several states and institutions represented by multiple studies. California and New York were particularly represented, including multiple studies conducted within the University of California system and the City University of New York system. Several student populations were also underrepresented. Although some studies focused on specific groups such as first-generation students, international students, student-athletes, veterans, medical students, and racial/ethnic minority students, relatively little research has examined food insecurity among graduate students, students with disabilities, Indigenous students, and refugees. Future research should prioritize populations that may face unique structural and socioeconomic barriers to food security and academic success.

### Limited longitudinal and intervention research

Most of the evidence identified in this review was derived from cross-sectional studies. Although these studies consistently demonstrated associations between food insecurity and poor academic outcomes, their design limits the ability to establish temporal relationships or causal pathways. Only a small number of longitudinal studies were identified, all conducted in the United States. Although these studies provide strong evidence that food insecurity predicts lower GPA, reduced educational attainment, and poor long-term socioeconomic outcomes, longitudinal evidence remains limited. Furthermore, existing intervention-related evidence was largely restricted to observational evaluations of campus food pantries or food assistance programs. Intervention studies, including randomized controlled trials and natural experiments, are needed to determine whether reducing food insecurity leads to measurable improvements in academic performance, retention, and graduation outcomes.

### Limited use of objective academic outcomes

Another important gap concerns the measurement of academic performance. Most studies relied on self-reported GPA, CGPA, grades, or perceived academic performance, which may be subject to recall bias and social desirability bias. Although several studies incorporated objective academic records, such as institutionally verified GPA, WAM, credit completion, retention, and graduation outcomes, these represented a minority of the literature. Future research would benefit from incorporating a broader range of objective academic indicators, including academic probation, course withdrawal, credit accumulation, retention, time to degree completion, and graduation rates.

#### Mechanisms linking food insecurity and academic performance

Although many studies documented an association between food insecurity and academic outcomes, fewer explicitly examined the mechanisms underlying this relationship. The qualitative and mixed-methods literature suggests that impaired concentration, fatigue, psychological distress, competing financial demands, and work–study disagreement may play important roles. Several quantitative studies also identified anxiety, depression, psychological distress, and sleep quality as potential mediators [29], [35], [51], [67], [81], [93]. However, evidence regarding these pathways remains limited and fragmented. Future research should employ longitudinal and mediation analyses to better understand the biological, psychological, social, and economic mechanisms linking food insecurity to academic success.

## Discussion

This scoping review synthesized evidence from 86 studies examining the relationship between food insecurity and academic performance among postsecondary students across eleven countries. Across quantitative, qualitative, mixed-methods, and longitudinal studies, the findings consistently demonstrated that food insecurity is associated with poor academic outcomes. Students experiencing food insecurity generally reported lower GPA, lower CGPA, worse perceived academic performance, reduced concentration and engagement in coursework, increased course withdrawals and failures, delayed graduation, and lower educational attainment. Although a small number of studies reported null associations for GPA, the overall pattern of evidence strongly suggests that food insecurity represents a substantial barrier to academic success in higher education.

The evidence was particularly consistent among studies examining GPA and grades. Most quantitative studies reported that food-insecure students had significantly lower GPAs or were less likely to achieve high academic standing than their food-secure peers. Longitudinal studies further strengthened this evidence by demonstrating that food insecurity predicted lower future GPA, reduced college completion, lower likelihood of obtaining a bachelor’s or graduate degree, and poor long-term socioeconomic outcomes. Beyond traditional academic indicators, the literature consistently demonstrated associations between food insecurity and broader measures of academic success. Food-insecure students were more likely to report difficulty concentrating, decreased classroom engagement, poor academic progress, lower confidence in academic achievement, increased absenteeism, course withdrawal, academic attrition, and anticipated delays in graduation. Qualitative studies further revealed that students frequently perceived food insecurity as directly interfering with their ability to learn, attend classes, complete assignments, and perform well on examinations.

The findings of this review suggest that the relationship between food insecurity and academic performance is likely multifactorial. Several quantitative studies identified psychological distress, anxiety, depression, and sleep disturbances as important mediating pathways between food insecurity and academic outcomes. For example, mediation analyses conducted in Malaysia, Australia, and the United States found that anxiety, depression, psychological distress, and poor mental health partially or fully explained the association between food insecurity and lower academic performance. These findings align with broader evidence demonstrating strong associations between food insecurity and adverse mental health outcomes among college students [113], [114], [115].

Qualitative evidence provided further insight into these pathways. Students consistently described persistent worry about obtaining food, managing finances, and meeting basic needs. These concerns frequently competed with academic demands, leading to difficulty concentrating during lectures, studying for examinations, and completing coursework. Many students reported being preoccupied with food acquisition, budgeting, or employment responsibilities rather than academic tasks. Such findings support theoretical models suggesting that unmet basic needs consume cognitive and emotional resources that would otherwise be available for learning and academic engagement.

Physiological consequences of food insecurity also emerged as important mechanisms. Students commonly described fatigue, hunger, headaches, low energy levels, poor sleep, and reduced stamina resulting from inadequate food intake or poor diet quality. These symptoms were repeatedly linked to impaired concentration, reduced classroom participation, and diminished academic productivity. Similar mechanisms have been observed in broader educational settings, where food insecurity has been associated with increased absenteeism and reduced academic engagement [27].

Another recurring theme involved competing financial demands and time constraints. Many students reported working additional hours to afford food, housing, tuition, and other necessities. This often resulted in missed classes, reduced study time, and lower academic engagement. Qualitative studies illustrated how students routinely made difficult trade-offs between purchasing food, paying for transportation, buying textbooks, and meeting other educational expenses. Such findings suggest that food insecurity should be viewed within a broader context of financial hardship and basic needs insecurity that may collectively undermine academic success.

Across the included studies, a recurring conceptual pathway emerged linking food insecurity to poorer academic outcomes through multiple intermediate mechanisms. Food insecurity was frequently associated with hunger, financial strain, inadequate nutrition, and psychological distress, which in turn were linked to depression and anxiety symptoms, sleep disturbances, fatigue, and reduced cognitive functioning. These factors commonly manifested as impaired concentration, decreased class attendance and engagement, and difficulty completing academic work, ultimately contributing to poorer academic outcomes, including lower GPA, course withdrawal or failure, reduced retention, and delayed graduation (Figure 3). The proposed conceptual framework is intended to summarize the pathways identified across the included literature and should not be interpreted as evidence of causality.

**FIGURE 3:**
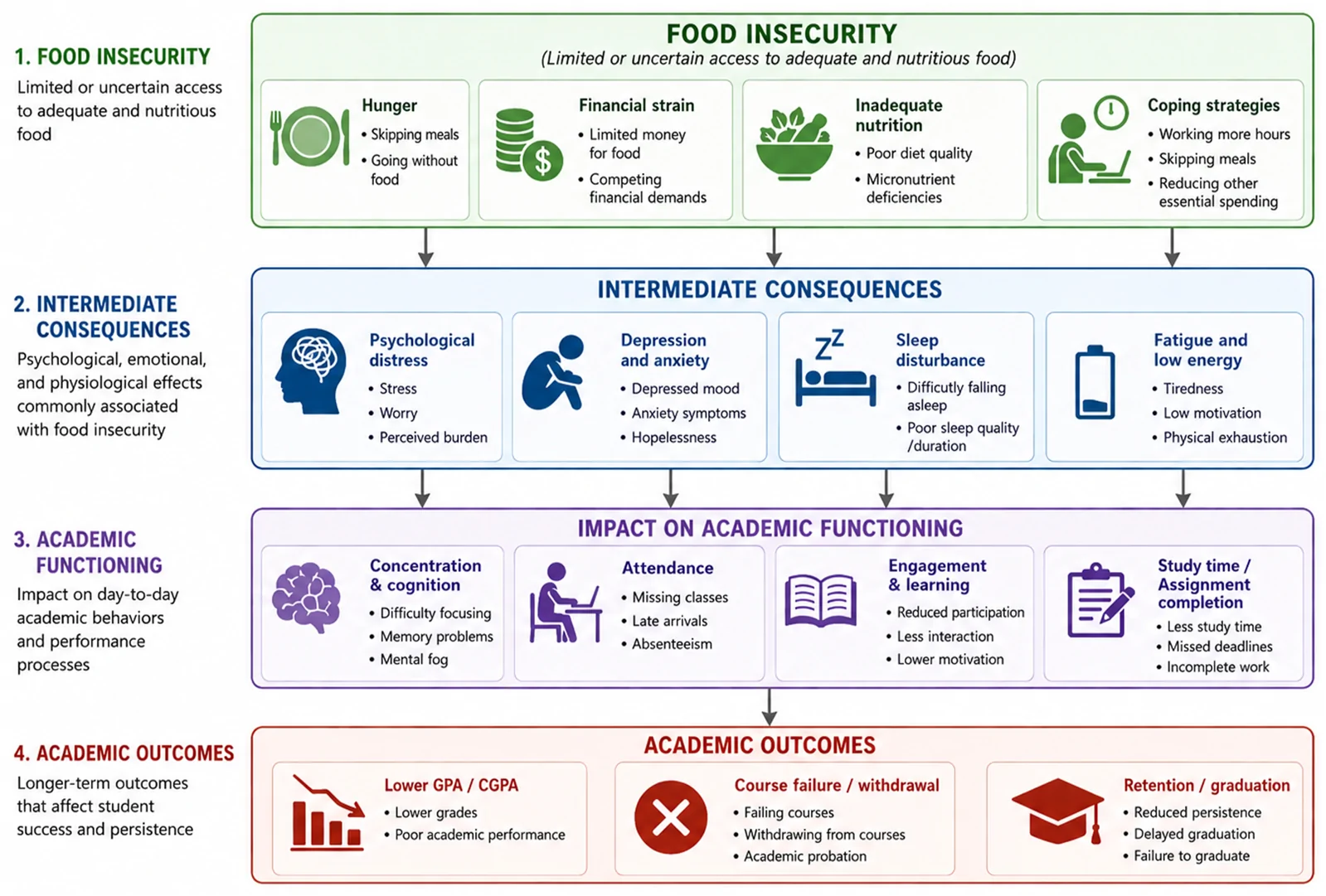
Conceptual framework summarizing pathways through which food insecurity may influence academic performance among university students, synthesized from the included studies. This figure was created with assistance from ChatGPT (OpenAI). CGPA, cumulative grade point average; GPA, grade point average.

### Implications for higher education institutions and policy

These findings have important implications for universities, colleges, and policymakers. Given the consistent evidence linking food insecurity with poor academic outcomes, addressing food insecurity should be considered not only a student welfare issue but also an educational success and retention strategy. Institutions invest substantial resources in student recruitment, retention, and graduation initiatives; however, these efforts may be undermined when students’ basic nutritional needs remain unmet.

Many institutions have responded by establishing campus food pantries, gardens, food rescue programs, and other basic needs programs [116]. A scoping review of postsecondary food security interventions in North America identified 4 published studies, all conducted in the United States, including programs at the University of Texas at San Antonio, La Salle University, and Appalachian State University, as well as a multi-institutional initiative [116]. The populations reached varied considerably; for example, participants in 1 program were predominantly Hispanic (37%) and African American (22%), with 46% being first-generation students, whereas another intervention included a predominantly white student population (92%). Emerging evidence suggests that these resources play an important role in supporting students experiencing food insecurity. For example, Esaryk et al. [117] found that campus food pantries provided critical emergency food assistance to students at elevated risk of food insecurity, particularly first-generation students and those experiencing financial hardship. Similarly, Waity et al. [118] reported that evidence of widespread food insecurity on campus prompted the development of institutional food pantry initiatives designed to support student well-being and academic success.

Despite the increasing availability of these services, utilization remains suboptimal. El Zein et al. [60] reported that although more than half of students were aware of campus food pantries, only approximately one-fifth of food-insecure students used them. Likewise, Cornett [119] found that awareness and use of campus food pantries were limited, with >40% of students attending institutions with food distribution centers reporting knowledge of these resources, along with low use by food-insecure students . Qualitative studies suggest that stigma, lack of awareness, perceptions that others were more deserving of assistance, and concerns regarding social judgment may contribute to underutilization [31], [32], [100], [102], [103].

These findings indicate that simply establishing food assistance programs may be insufficient. Institutions should consider comprehensive approaches that combine food access initiatives with outreach, education, and stigma-reduction strategies. Improving awareness of available resources, integrating food security screening into student support services, and strengthening referral pathways may help increase utilization among students most in need. Furthermore, recent evidence suggests that interventions extending beyond emergency food assistance may improve educational outcomes. In 1 randomized controlled trial, students offered campus meal vouchers completed more credits and were more likely to graduate than comparable peers who did not receive the intervention [120]. Such findings highlight the potential academic benefits of addressing food insecurity through structured institutional support.

At the policy level, barriers to participation in federal nutrition assistance programs remain an important concern. Research suggests that many students are unaware of program eligibility criteria and allowable benefits, potentially limiting enrollment in programs such as SNAP [121]. Enhancing outreach, simplifying eligibility processes, and expanding awareness of available supports may represent additional opportunities to improve food security among postsecondary students.

### Strengths and limitations

This scoping review has several strengths. To our knowledge, it is among the most comprehensive reviews to synthesize evidence on the relationship between food insecurity and academic performance among students in higher education. The review employed a broad search strategy across multiple databases and included quantitative, qualitative, mixed-methods, and longitudinal studies, which resulted in a comprehensive examination of both the magnitude of the association and the mechanisms through which food insecurity may influence academic outcomes. In addition, the use of a scoping review methodology was appropriate given the heterogeneity of study designs, outcome measures, and varied conceptualizations of academic performance across the literature. The review also captured diverse student populations across multiple geographic and institutional contexts, including undergraduate, graduate, and professional students, providing a broader understanding of how food insecurity may affect academic outcomes across different postsecondary settings.

Several limitations of this scoping review should also be acknowledged. First, although a comprehensive search strategy was employed across multiple databases, it is possible that relevant studies were missed, particularly those published in sources not indexed in the selected databases. Second, the review was limited to studies published in English, which may have resulted in the exclusion of relevant evidence published in other languages. Third, consistent with the objectives of a scoping review, the focus was on mapping and synthesizing the breadth of available evidence rather than critically appraising study quality or conducting a quantitative synthesis. Finally, the considerable heterogeneity in study designs, measures of food insecurity, and indicators of academic performance limited direct comparisons across studies and precluded the identification of standardized effect estimates.

In conclusion, this scoping review demonstrates a consistent and substantial body of evidence linking food insecurity with poor academic outcomes among postsecondary students. Across study designs and educational settings, food insecurity was associated with lower GPA, reduced academic achievement, impaired concentration, decreased class attendance and engagement, greater course withdrawal and failure, delayed graduation, and lower educational attainment. Qualitative and mixed-methods studies further revealed that these associations are likely driven by multiple interconnected pathways, including hunger, fatigue, psychological distress, sleep disruption, competing financial demands, and work–study disagreement.

Taken together, the findings suggest that food insecurity is not solely a nutrition or financial concern but also an important educational issue that may undermine students’ ability to succeed in higher education. As colleges and universities increasingly seek to improve student retention, persistence, and graduation outcomes, addressing food insecurity should be a critical component of student success initiatives [122], [123]. Expanding access to effective food assistance programs, reducing barriers to program utilization (e.g., decreasing stigmatization), strengthening institutional support systems, and improving access to public nutrition assistance programs such as SNAP may help mitigate the academic consequences of food insecurity [124], [125].

Despite the growing literature, important knowledge gaps remain regarding causal pathways, long-term educational impacts, and the effectiveness of interventions designed to improve food security among postsecondary students. Future research should prioritize longitudinal and intervention studies, incorporate objective academic outcomes, and expand investigation into underrepresented populations and geographic regions [126], [127]. For example, recent evidence suggests that minority students in the United States, particularly African American and Hispanic students, experience a higher prevalence of food insecurity and may be more likely to experience poorer academic outcomes [126]. Strengthening the evidence base in these areas will be essential for informing policies and programs aimed at promoting both food security and academic success among postsecondary students.

## Author contributions

The authors’ responsibilities were as follows – MS, GL: designed the research; EA, AM, MHL, MS: conducted the research and wrote the article; GL: performed the study screening and selection and critically revised the manuscript; MS: took primary responsibility for the final content; and all authors: read and approved the final manuscript.

## Declaration of Generative AI and AI-assisted technologies in the writing process

During the preparation of this work, the authors used ChatGPT solely to improve the readability and language of portions of the manuscript. After using this tool, the authors reviewed and edited the content as needed and took full responsibility for the content of the publication. ChatGPT was also used to assist in the visual design of the conceptual framework (Figure 3).

## Funding

None.

## Conflict of interest

The authors report no conflicts of interest.
