# Supplementary material for: Food Insecurity and Academic Performance among Postsecondary Students: A Global Scoping Review
Source: Curr Dev Nutr. 2026 Oct 6;10(11):109551. doi: 10.1016/j.cdnut.2026.109551 (PMC13722069; doi:10.1016/j.cdnut.2026.109551)
Supplement: Supplementary file 1 [file mmc1.docx]

**Supplementary Table 1:** MEDLINE Search Strategy for Food Insecurity and Academic Outcomes Among Higher Education Students

| 1. | Food Insecurity.mp. or Food Supply/ or Food Insecurity/ or Diet/ or Malnutrition/ or Poverty/ |  |
| --- | --- | --- |
| 2. | limit 1 to english language |  |
| 3. | "food security".mp. [mp=title, book title, abstract, original title, name of substance word, subject heading word, floating sub-heading word, keyword heading word, organism supplementary concept word, protocol supplementary concept word, rare disease supplementary concept word, unique identifier, synonyms, population supplementary concept word, anatomy supplementary concept word] |  |
| 4. | limit 3 to english language |  |
| 5. | "food insufficiency".mp. [mp=title, book title, abstract, original title, name of substance word, subject heading word, floating sub-heading word, keyword heading word, organism supplementary concept word, protocol supplementary concept word, rare disease supplementary concept word, unique identifier, synonyms, population supplementary concept word, anatomy supplementary concept word] |  |
| 6. | limit 5 to english language |  |
| 7. | "nutritional insecurity".mp. [mp=title, book title, abstract, original title, name of substance word, subject heading word, floating sub-heading word, keyword heading word, organism supplementary concept word, protocol supplementary concept word, rare disease supplementary concept word, unique identifier, synonyms, population supplementary concept word, anatomy supplementary concept word] |  |
| 8. | limit 7 to english language |  |
| 9. | hunger.mp. [mp=title, book title, abstract, original title, name of substance word, subject heading word, floating sub-heading word, keyword heading word, organism supplementary concept word, protocol supplementary concept word, rare disease supplementary concept word, unique identifier, synonyms, population supplementary concept word, anatomy supplementary concept word] |  |
| 10. | limit 9 to english language |  |
| 11. | "academic persistence".mp. [mp=title, book title, abstract, original title, name of substance word, subject heading word, floating sub-heading word, keyword heading word, organism supplementary concept word, protocol supplementary concept word, rare disease supplementary concept word, unique identifier, synonyms, population supplementary concept word, anatomy supplementary concept word] |  |
| 12. | limit 11 to english language |  |
| 13. | "degree completion".mp. [mp=title, book title, abstract, original title, name of substance word, subject heading word, floating sub-heading word, keyword heading word, organism supplementary concept word, protocol supplementary concept word, rare disease supplementary concept word, unique identifier, synonyms, population supplementary concept word, anatomy supplementary concept word] |  |
| 14. | limit 13 to english language |  |
| 15. | "educational attainment".mp. [mp=title, book title, abstract, original title, name of substance word, subject heading word, floating sub-heading word, keyword heading word, organism supplementary concept word, protocol supplementary concept word, rare disease supplementary concept word, unique identifier, synonyms, population supplementary concept word, anatomy supplementary concept word] |  |
| 16. | limit 15 to english language |  |
| 17. | Academic Performance.mp. or Achievement/ or Academic Performance/ or Educational Status/ |  |
| 18. | limit 17 to english language |  |
| 19. | Academic Achievement.mp. or Academic Success/ |  |
| 20. | limit 19 to english language |  |
| 21. | GPA.mp. [mp=title, book title, abstract, original title, name of substance word, subject heading word, floating sub-heading word, keyword heading word, organism supplementary concept word, protocol supplementary concept word, rare disease supplementary concept word, unique identifier, synonyms, population supplementary concept word, anatomy supplementary concept word] |  |
| 22. | limit 21 to english language |  |
| 23. | grades.mp. [mp=title, book title, abstract, original title, name of substance word, subject heading word, floating sub-heading word, keyword heading word, organism supplementary concept word, protocol supplementary concept word, rare disease supplementary concept word, unique identifier, synonyms, population supplementary concept word, anatomy supplementary concept word] |  |
| 24. | limit 23 to english language |  |
| 25. | "learning outcome".mp. [mp=title, book title, abstract, original title, name of substance word, subject heading word, floating sub-heading word, keyword heading word, organism supplementary concept word, protocol supplementary concept word, rare disease supplementary concept word, unique identifier, synonyms, population supplementary concept word, anatomy supplementary concept word] |  |
| 26. | limit 25 to english language |  |
| 27. | "educational outcome".mp. [mp=title, book title, abstract, original title, name of substance word, subject heading word, floating sub-heading word, keywordheading word, organism supplementary concept word, protocol supplementary concept word, rare disease supplementary concept word, unique identifier,synonyms, population supplementary concept word, anatomy supplementary concept word] | |
| 28. | limit 27 to english language | |
| 29. | "educational status".mp. [mp=title, book title, abstract, original title, name of substance word, subject heading word, floating sub-heading word, keywordheading word, organism supplementary concept word, protocol supplementary concept word, rare disease supplementary concept word, unique identifier,synonyms, population supplementary concept word, anatomy supplementary concept word] | |
| 30. | limit 29 to english language | |
| 31. | "grade point average".mp. [mp=title, book title, abstract, original title, name of substance word, subject heading word, floating sub-heading word, keywordheading word, organism supplementary concept word, protocol supplementary concept word, rare disease supplementary concept word, unique identifier,synonyms, population supplementary concept word, anatomy supplementary concept word] | |
| 32. | limit 31 to english language | |
| 33. | 2 or 4 or 6 or 8 or 10 | |
| 34. | 12 or 14 or 16 or 18 or 20 or 22 or 24 or 26 or 28 or 30 or 32 | |
| 35. | Students, Premedical/ or Students, Medical/ or Students, Public Health/ or Students, Health Occupations/ or Students, Dental/ or Students, Nursing/ orStudents/ or Students, Pharmacy/ | |
| 36. | limit 35 to english language | |
| 37. | "higher education".mp. [mp=title, book title, abstract, original title, name of substance word, subject heading word, floating sub-heading word, keywordheading word, organism supplementary concept word, protocol supplementary concept word, rare disease supplementary concept word, unique identifier,synonyms, population supplementary concept word, anatomy supplementary concept word] | |
| 38. | limit 37 to english language | |
| 39. | college.mp. [mp=title, book title, abstract, original title, name of substance word, subject heading word, floating sub-heading word, keyword heading word,organism supplementary concept word, protocol supplementary concept word, rare disease supplementary concept word, unique identifier, synonyms,population supplementary concept word, anatomy supplementary concept word] | |
| 40. | limit 39 to english language | |
| 41. | university.mp. [mp=title, book title, abstract, original title, name of substance word, subject heading word, floating sub-heading word, keyword headingword, organism supplementary concept word, protocol supplementary concept word, rare disease supplementary concept word, unique identifier,synonyms, population supplementary concept word, anatomy supplementary concept word] | |
| 42. | undergraduate.mp. [mp=title, book title, abstract, original title, name of substance word, subject heading word, floating sub-heading word, keyword headingword, organism supplementary concept word, protocol supplementary concept word, rare disease supplementary concept word, unique identifier,synonyms, population supplementary concept word, anatomy supplementary concept word] | |
| 43. | limit 42 to english language | |
| 44. | postsecondary.mp. [mp=title, book title, abstract, original title, name of substance word, subject heading word, floating sub-heading word, keyword headingword, organism supplementary concept word, protocol supplementary concept word, rare disease supplementary concept word, unique identifier,synonyms, population supplementary concept word, anatomy supplementary concept word] | |
| 45. | limit 44 to english language | |
| 46. | graduate.mp. [mp=title, book title, abstract, original title, name of substance word, subject heading word, floating sub-heading word, keyword headingword, organism supplementary concept word, protocol supplementary concept word, rare disease supplementary concept word, unique identifier,synonyms, population supplementary concept word, anatomy supplementary concept word] | |
| 47. | limit 46 to english language | |
| 48. | student*.mp. [mp=title, book title, abstract, original title, name of substance word, subject heading word, floating sub-heading word, keyword heading word,organism supplementary concept word, protocol supplementary concept word, rare disease supplementary concept word, unique identifier, synonyms,population supplementary concept word, anatomy supplementary concept word] | |
| 49. | limit 48 to english language | |
| 50. | "tertiary education".mp. [mp=title, book title, abstract, original title, name of substance word, subject heading word, floating sub-heading word, keywordheading word, organism supplementary concept word, protocol supplementary concept word, rare disease supplementary concept word, unique identifier,synonyms, population supplementary concept word, anatomy supplementary concept word] | |
| 51. | limit 50 to english language | |
| 52. | 36 or 38 or 40 or 41 or 43 or 45 or 47 or 49 or 51 | |
| 53. | 33 and 34 and 52 | |
| 54. | 36 or 38 or 40 or 42 or 43 or 45 or 47 or 51 | |
| 55. | 33 and 34 and 54 | |
